# Distinct Defence Mechanisms of Allelopathic Rice Against Quinclorac‐Susceptible and ‐Resistant Barnyardgrass: Involvement of Specific Metabolites and Rhizosheath Microbiota

**DOI:** 10.1111/pbi.70611

**Published:** 2026-02-25

**Authors:** Shuyan Li, Qiling Yan, Jianhua Tong, Yongfeng Li, Lianyang Bai, Qiong Peng

**Affiliations:** ^1^ Hunan Institute of Plant Protection Hunan Academy of Agricultural Sciences Changsha China; ^2^ Yuelushan Laboratory Changsha China; ^3^ School of Biological Sciences University of Western Australia Perth Western Australia Australia; ^4^ College of Bioscience and Technology Hunan Agricultural University Changsha China; ^5^ Institute of Germplasm Resources and Biotechnology Jiangsu Academy of Agricultural Sciences Nanjing China

**Keywords:** allelochemicals, herbicidal activity, nitrogen utilisation

## Abstract

Allelopathic rice is increasingly recognised as a promising strategy for sustainable weed management. Resistance to the herbicide quinclorac is widespread in barnyardgrass, but it remains unclear whether allelopathic rice exerts the same defence against herbicide‐susceptible and ‐resistant barnyardgrass. We conducted integrated transcriptomic, metabolomic, and metagenomic analyses to investigate the responses of allelopathic rice to quinclorac‐susceptible (S) and ‐resistant barnyardgrass (R) lines. Distinct chemical strategies were identified in allelopathic rice: Terpenoids (e.g., carnosic acid, phytocassane B, and ipomeatetrahydrofuran) mainly suppressed S, while amino acids (e.g., pipecolic acid, L‐glutamate, and L‐histidine) were key against R. Correspondingly, terpenoid biosynthesis and nitrogen metabolism were the most enriched pathways under S and R stress, respectively. Additionally, terpenoid accumulation correlated positively with salicylic acid (SA) and jasmonic acid (JA) concentrations in roots under S. Both terpenoids and amino acids formed the stable ecological networks with rhizosheath microbiota. Functional metagenomic analysis further showed that ABC transporter and quorum sensing pathways were upregulated under S, whereas nitrogen fixation predominated under R. Notably, amino acids formed a nitrogen‐related ecological network with nitrogen‐metabolising microbiota, contributing to improved plant‐available soil nitrogen and total nitrogen content in rice plants. Bioassays showed that exogenous pipecolic acid (≥ 40 μM) and L‐histidine (80 μM) inhibited barnyardgrass seedling growth without affecting allelopathic and non‐allelopathic rice. These findings demonstrate that allelopathic rice employs divergent chemical‐microbial defence strategies against S and R barnyardgrass, highlight the dual role of amino acids, and provide a basis for precision weed management, particularly for herbicide‐resistant weeds in paddy fields.

## 1 Introduction

Rice ( *Oryza sativa* L.)‐infesting weeds pose a significant threat to rice production and food safety. Barnyardgrass ( *Echinochloa crus‐galli* L.) is a prevalent and highly damaging weed species in rice paddock worldwide (Aoki and Yamaguchi 2008), causing rice yield losses ranging from 21% to 79% (Bajwa et al. 2015; Zhang et al. 2017, 2021). Synthetic chemical herbicides are primary tools for weed control. However, over‐reliance on herbicides has led to the evolution of herbicide resistance in *E. crus‐galli* (Heap 2024; Peng et al. 2019) and has caused environmental and health problems. All these concerning issues underline an urgent need for alternative strategies for sustainable weed management in paddy systems.

Allelopathy refers to the biological process in which one organism affects the growth, survival, and reproduction of another through the release of allelochemicals (Cheng and Cheng 2015). In crop‐weed interactions, this process provides cultivated plants with a competitive advantage by inhibiting surrounding weeds (Zheng et al. 2015). Various classes of secondary metabolites, including phenolics, terpenoids, and nitrogen‐containing compounds, have been identified as allelochemicals (Kong et al. 2019; Macías et al. 2019), functioning as “allelopathic weapons” that inhibit the growth of neighbouring plant species (Vivanco et al. 2004). It was reported that allelopathic rice reduced barnyardgrass root length, total root area, maximum root width and depth by secreting allelochemicals, including phenolic acid, flavones, and terpenoids (Kong et al. 2019; Zhang et al. 2017), showing the ability of allelopathic rice to suppress cooccurring barnyardgrass growth. Interestingly, allelopathic rice has a stronger inhibition for resistant barnyardgrass than for susceptible barnyardgrass (Yang et al. 2017). Candidate genes (e.g., *CYP99A2*, *osMAS*, and *PAL*) responsible for regulating the biosynthesis of these secondary metabolites with barnyardgrass‐suppressing activity in allelopathic rice have been widely studied (Shimura et al. 2007; Sultana et al. 2023; Wang et al. 2011). In addition, increasing evidence demonstrates that plants adapt to biotic stress (such as pathogen attacks or insect herbivory) by changing plant hormone levels, especially salicylic acid (SA) and jasmonic acid (JA), that induces the terpenoid synthase gene expression (Li et al. 2016; You et al. 2011). However, knowledge about the interactions between allelochemical biosynthesis and the plant defence system is still sparse.

Plant roots are naturally exposed and respond to diverse stresses by exuding a variety of metabolites, which modulate the assembly dynamics of root‐associated microbiome to mitigate the encountered stress (Berendsen et al. 2018; Carrión et al. 2019; Dudenhöffer et al. 2016; Liu et al. 2021). Various beneficial rhizosphere microbes, including *Pseudomonas*, *Bacillus*, and *Acinetobacter* (Pieterse et al. 2014), can alleviate the adverse stress effects on plant growth by enhancing nutrient acquisition (Trivedi et al. 2020), activating induced systemic resistance (Pieterse et al. 2014; Pineda et al. 2010) and defence regulatory genes (Pineda et al. 2010). Increasing evidences demonstrate that crops including wheat ( *Triticum aestivum* L.) (Hu et al. 2023), barley ( *Hordeum vulgare* L.) (Jousset et al. 2011), and sorghum ( *Sorghum bicolor* (L.) Moench) (Lendzemo et al. 2007) adapt to biotic stress (e.g., pathogen or weed stress) by changing root specific metabolites in a way that favours the recruitment of plant‐beneficial microorganisms. Early studies revealed that the production of allelochemicals in rice roots, induced by barnyardgrass infestation, could provide carbon source and stimulate soil bacteria (Bacilio‐Jiménez et al. 2003; Kong et al. 2004). For example, the exudation of phenolic acids by allelopathic rice roots has been shown to significantly increase the abundance of *Myxococcus* in the rhizosphere (Li, Jian, et al. 2020). Despite extensive documentation of beneficial microbiome assembly driven by root specific metabolites, the interplay between root metabolite accumulation and rhizosheath microbiome recruitment in allelopathic rice, particularly their synergistic effects on weed suppression remain poorly elucidated.

In our previous study, we observed that the flowering time and net photosynthetic rate of the quinclorac‐resistant (R) barnyardgrass differ significantly from those of the susceptible (S) barnyardgrass (Li, Wang, et al. 2020; Figure S1). We wonder if these differences in R and S barnyardgrass can lead to differential interactions with allelopathic rice? To justify this, we (1) profiled the specific allelochemicals and the related genes in allelopathic rice against quinclorac‐susceptible and ‐resistant barnyardgrass; (2) evaluated the establishment of beneficial rhizosheath microbiome in allelopathic rice to alleviate different barnyardgrass stresses; and (3) tested the inhibitory activity of candidate allelopathic metabolites on barnyardgrass growth and their safety to rice seedlings. This study aims to establish a theoretical framework for the crosstalk between rice roots, root exudates, and beneficial rhizosphere microbiota, providing insights for novel ecological control strategies supporting sustainable agricultural practices, particularly in addressing herbicide resistance challenges in paddy ecosystems.

## 2 Results

### 2.1 Allelopathic Rice Responds Differently to Quinclorac‐Resistant and ‐Susceptible Barnyardgrass in Net Photosynthetic Rate

Leaf photosynthetic rates in allelopathic rice varied significantly when monocultured (PI) or cultured with quinclorac‐resistant (PIR) and ‐susceptible barnyardgrass (PIS) (Table 1). In the PIR treatment, the net photosynthetic rate (*Pn*), transpiration rate (*Tr*) (*F*_2,15_ = 2.09, *p* = 0.16), intercellular CO_2_ concentration (Ci) (*F*_2,15_ = 1.41, *p* = 0.28), and stomatal conductance (*gs*) (*F*_2,15_ = 1.18, *p* = 0.33) in allelopathic rice were slightly but insignificantly reduced compared to ‘PI312777’ monoculture (PI); except, there was a significant (*F*_2,15_ = 11.07, *p* = 0.001) increase (20%, 3.48 μmol m^−2^ s^−1^) in net *Pn* in PIS relative to PI (95% CI: 0.4–6.6; Table 1). The lowest *Ci* was found in PIS, which was 17% lower than PI (Table 1). The flag leaf thickness in allelopathic rice tended to increase under barnyardgrass stress. Specifically, relative to the control, leaf thickness in PIR increased by 24%. Thus, quinclorac‐resistant barnyardgrass seemed to suppress, and quinclorac‐susceptible biotype enhance the *Pn* in allelopathic rice.

**TABLE 1:** Leaf photosynthetic parameters, and flag leaf thickness, in the allelopathic rice under the three treatments: Monocultured allelopathic rice (PI) with no barnyardgrass, allelopathic rice co‐cultured with quinclorac‐resistant (PIR) and ‐susceptible (PIS) barnyardgrass. Data are mean ± SD (*n* = 6). Different letters within a row indicate significant differences between the treatments (*p* < 0.05) based on one‐way ANOVA followed by Tukey's HSD test.

	PI	PIR	PIS
Net photosynthetic rate (μmol m^−2^ s^−1^)	15 ± 2.8 b	13 ± 2.3 b	18 ± 1 a
Transpiration rate (mmol m^−2^ s^−1^)	3.9 ± 1.4 a	2.5 ± 0.8 a	3.3 ± 1.1 a
Intercellular CO_2_ concentration (μmol mol^−1^)	254 ± 36 a	238 ± 36 a	210 ± 61 a
Stomatal conductance (mol m^−2^ s^−1^)	0.21 ± 0.08 a	0.15 ± 0.05 a	0.19 ± 0.07 a
Leaf thickness (μm)	128 ± 7.7 b	159 ± 5.3 a	129 ± 6.4 b

### 2.2 Quinclorac‐Resistant and ‐Susceptible Barnyardgrass Induce Differential Soil Physicochemical Property Changes

No significant differences in soil pH (*F*_2,6_ = 3.2, *p* = 0.11), TOM (total organic matter) concentration (*F*_2,6_ = 2.62, *p* = 0.15), AK (available potassium) (*F*_2,6_ = 0.09, *p* = 0.91), CEC (cation exchange capacity; *F*_2,6_ = 1.55, *p* = 0.29) and EC (electrical conductivity; *F*_2,6_ = 1.16, *p* = 0.38) among PI, PIR and PIS were observed (Table 2). However, barnyardgrass stresses reduced AP (available phosphorus) by 7% in PIR and 10% (*F*_2,6_ = 8.46, *p* = 0.02) in PIS compared with the control. Soil TOM (total organic matter) concentration showed a particularly increase (16%) in the PIR treatment. The soil electrical conductivity (EC) increased by 7% in the PIR treatment; however, this change was not statistically significant. The concentration of plant‐available soil nitrogen (AN) differed significantly among treatments (*F*_2,15_ = 15.12, *p* = 2.54e‐04), increasing in the PIR treatment relative to PI by 6 mg kg^−1^ (95% CI: 2.95–9.05), whereas no significant change was observed in PIS. Interestingly, the total N concentration in allelopathic rice was significantly (*F*_2,15_ = 53.36, *p* = 1.52e‐07) increased by 4.4 mg g^−1^ in PIR compared with PI (95% CI: 3.3–5.5), suggesting that stress from resistant‐barnyardgrass can induce soil nitrogen accumulation in allelopathic rice.

**TABLE 2:** Soil physicochemical properties in the allelopathic rice under the three treatments (PI, PIR and PIS) TOM: Total organic matter, AN: Plant‐available soil nitrogen, AP: Available phosphorus, AK: Available potassium, CEC: Cation exchange capacity, EC: Electrical conductivity, TN: Total nitrogen concentration. Data are mean ± SD. Different letters within a row indicate significant differences between the treatments (*p* < 0.05), based on one‐way ANOVA followed by Tukey's HSD test.

	PI	PIR	PIS
pH	6.3 ± 0.09 a	6.4 ± 0.03 a	6.4 ± 0.04 a
TOM (g kg^−1^)	32 ± 3.2 a	37 ± 2.8 a	34 ± 1.9 a
AN (mg kg^−1^)	76 ± 1.9 b	82 ± 1.6 a	76 ± 2.8 b
AP (mg kg^−1^)	10 ± 0.4 a	9.3 ± 0.4 ab	9 ± 0.2 b
AK (mg kg^−1^)	160 ± 6 a	157 ± 13 a	160 ± 13 a
CEC (cmol kg^−1^)	8.4 ± 0.3 a	8.8 ± 0.5 a	8.4 ± 0.2 a
EC (us cm^−1^)	41 ± 2.7 a	44 ± 6.3 a	39 ± 0.4 a
TN (mg g^−1^)	21 ± 0.5 b	25 ± 0.8 a	21 ± 1 b

### 2.3 Allelopathic Rice Roots Respond to ‐Resistant and ‐Susceptible Barnyardgrass Stress in Phytohormone Levels

Salicylic acid (SA) and jasmonic acid (JA) were assessed in the leaf and root tissue of allelopathic rice under ‐resistant and ‐susceptible barnyardgrass stress (Table 3). Rice leaf SA (*F*_2,6_ = 2.81, *p* = 0.14) and JA (*F*_2,6_ = 1.98, *p* = 0.22) showed no significant differences under barnyardgrass stress compared to the PI control. Root SA and JA concentrations increased in PIS relative to PI (SA: *F*_2,6_ = 70.24, *p* = 6.87e‐05; JA: *F*_2,6_ = 6.74, *p* = 0.03), corresponding to increases of 99 ng g^−1^ (95% CI: 59.3–138.6) and 225 ng g^−1^ (95% CI: 14.7–435.3), respectively. In contrast, root SA concentration decreased in the PIR treatment by 61.6 ng g^−1^ relative to PI (95% CI: −101.2 to −21.9). These results indicate that allelopathic rice roots exhibit a more pronounced response in the production of SA and JA compared with allelopathic rice leaves under barnyardgrass stress.

**TABLE 3:** Changes in main phytohormone levels in leaves and roots of the allelopathic rice under the PI, PIR and PIS treatments. SA, salicylic acid, JA, jasmonic acid. Data are mean ± SD (*n* = 3). Different letters within a row indicate significant differences between the treatments (*p* < 0.05), based on one‐way ANOVA followed by Tukey's HSD test.

Tissue	Treatment	SA (ng g^−1^)	JA (ng g^−1^)
Leaf	PI	16 001 ± 586 a	410 ± 85 a
	PIR	15 182 ± 1659 a	558 ± 135 a
	PIS	17 548 ± 1236 a	504 ± 14 a
Root	PI	220 ± 9 b	228 ± 19 b
	PIR	159 ± 7 c	218 ± 101 b
	PIS	319 ± 27 a	453 ± 115 a

### 2.4 Distinct Transcriptomic Alterations in Allelopathic Rice Roots in Response to ‐Resistant and ‐Susceptible Barnyardgrass Stress

**FIGURE 1:**
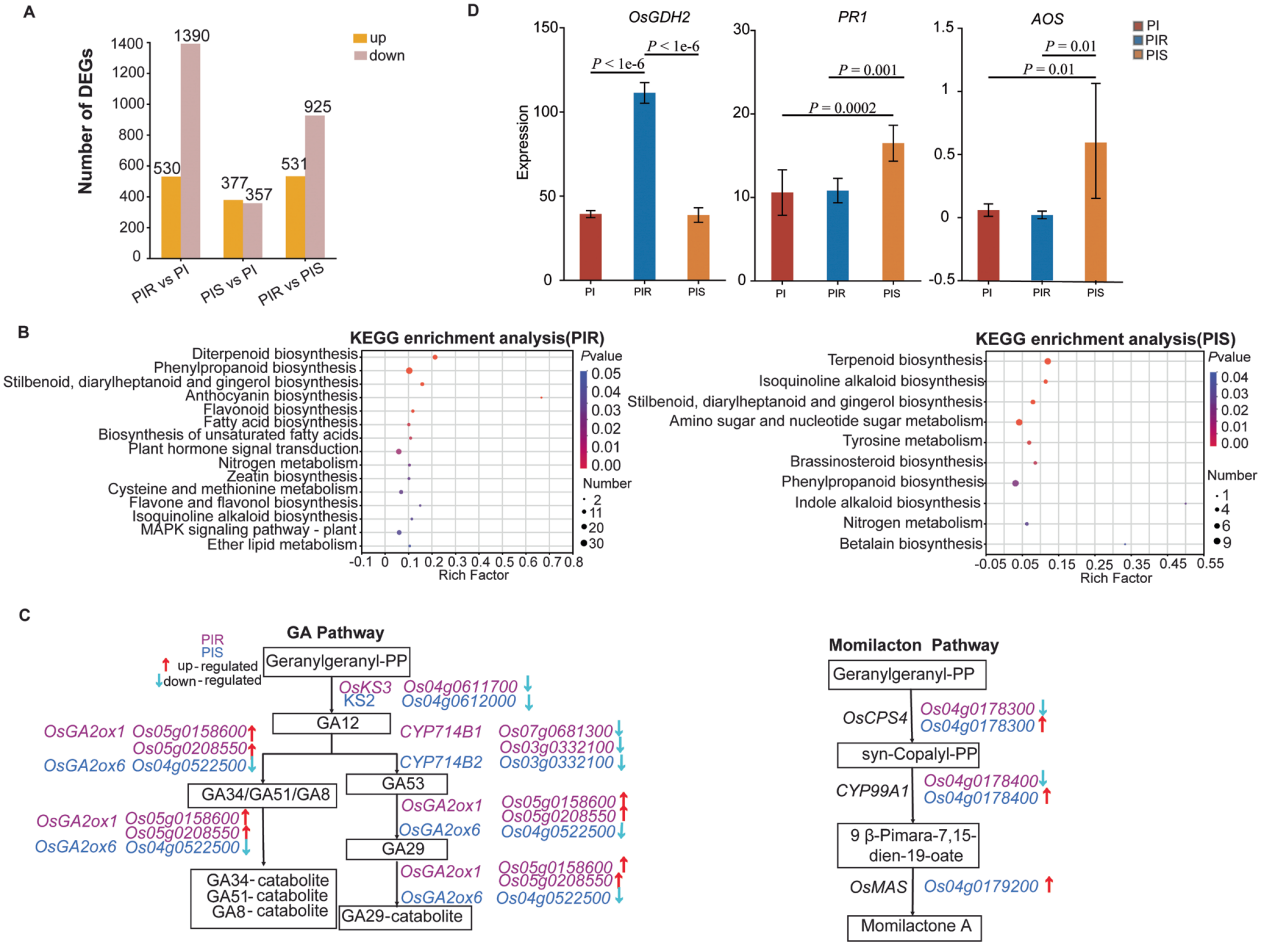
Differentially expressed genes (DEGs) in allelopathic rice root tissue under barnyardgrass stress. PI, PIR, and PIS represent monocultured allelopathic rice with no barnyardgrass, allelopathic rice co‐cultured with quinclorac‐resistant and ‐susceptible barnyardgrass, respectively. (A) The number of DEGs found in different treatments. (B) KEGG (Kyoto Encyclopedia of Genes and Genomes) enrichment analysis of DEGs in rice under PIR and PIS treatments. Significantly enriched KEGG pathways (*p* < 0.05) were identified using Fisher's exact test with Benjamini–Hochberg correction. The dot size represents the number of DEGs enriched in each pathway, and the colour indicates the *p*‐value. (C) DEGs related to GA (gibberellin acid) and momilactone pathways in allelopathic rice under barnyardgrass stress. Up‐regulated and down‐regulated genes are indicated by red and blue arrows, respectively. The purple and blue texts represent DEGs under the PIR and PIS treatments, respectively. (D) DEGs of *OsGDH2* (nitrogen metabolism), *PR1* (JA), and *AOS* (SA) in allelopathic rice under PIR and PIS treatments were identified using one‐way analysis of variance (ANOVA), followed by Tukey's honestly significant difference (HSD) post hoc test for pairwise comparisons. A 95% confidence interval was applied, and genes with *p* < 0.05 were considered significantly different.

High‐quality transcriptome sequencing yielded 125.20 Gb of clean data from 18 root samples, averaging 6.15 Gb per sample. The percentage of Q30 bases exceeded 94%, and the GC content was approximately 44%, with an error rate of 0.02% (Table S1). For barnyardgrass stress‐responsive DEGs, 1920 (530 up‐regulated and 1390 down‐regulated) and 734 (377 up‐regulated and 357 down‐regulated) were identified in PIR vs. PI and PIS vs. PI groups respectively (Figure 1A), indicating that allelopathic rice exhibited a more vigorous response of allelopathic rice at the transcriptome level under PIR.

A KEGG enrichment analysis was performed to search for the DEGs involved in metabolic or signal transduction pathways. Fifteen and ten significantly enriched pathways were identified in allelopathic rice under PIR and PIS treatments, respectively (Figure 1B). Intriguingly, PIS and PIR stress exerted opposite regulatory effects on some pathways. For example, in the diterpenoid biosynthesis pathway, *OsCPS4* (*LOC_Os04g0178300*) and *CYP99A1* (*LOC_Os04g0178400*) encoding key enzymes for momilactone biosynthesis displayed divergent expression patterns: up‐regulated (2.65‐ and 3.14‐fold, respectively) in PIS but down‐regulated in PIR (Figure 1C). In the GA biosynthesis pathway, *OsGA2ox1* (*LOC_Os05g0158600*, *LOC_Os05g0208550*) was up‐regulated (2.07‐ and 2.45‐fold, respectively) in PIR, whereas *OsGA2ox6* (*LOC_Os04g0522500*) was down‐regulated in PIS. For nitrogen metabolism, *OsGDH2* (*LOC_Os04g0543900*) was only upregulated (2.87‐fold) in PIR (Figure 1D). The expression levels of SA‐ and JA‐related genes *PR1* (*Os02g0786500*; *F*_2,15_ = 16.91, *p* = 0.0001) and *AOS* (*Os02g0218700*; *F*_2,15_ = 8.24, *p* = 0.004) were significantly (*p* < 0.05) upregulated (4.2‐ and 7.67‐fold, respectively) in allelopathic rice roots in PIS (Figure 1D). Furthermore, the results of quantitative real‐time PCR (qRT‐PCR) were consistent with the transcriptome data (Table S2).

Taken together, these results suggest that resistant and susceptible barnyardgrass had contrasting effects on rice root gene expression. Particularly, in the terpenoid biosynthesis pathway, *OsCPS4* and *CYP99A1* were upregulated in PIS but downregulated in PIR, which may cause differences in rice's response to barnyardgrass stress.

### 2.5 Allelopathic Rice Roots Secrete Different Allelochemicals in Response to ‐Resistant and ‐Susceptible Barnyardgrass Stress

**FIGURE 2:**
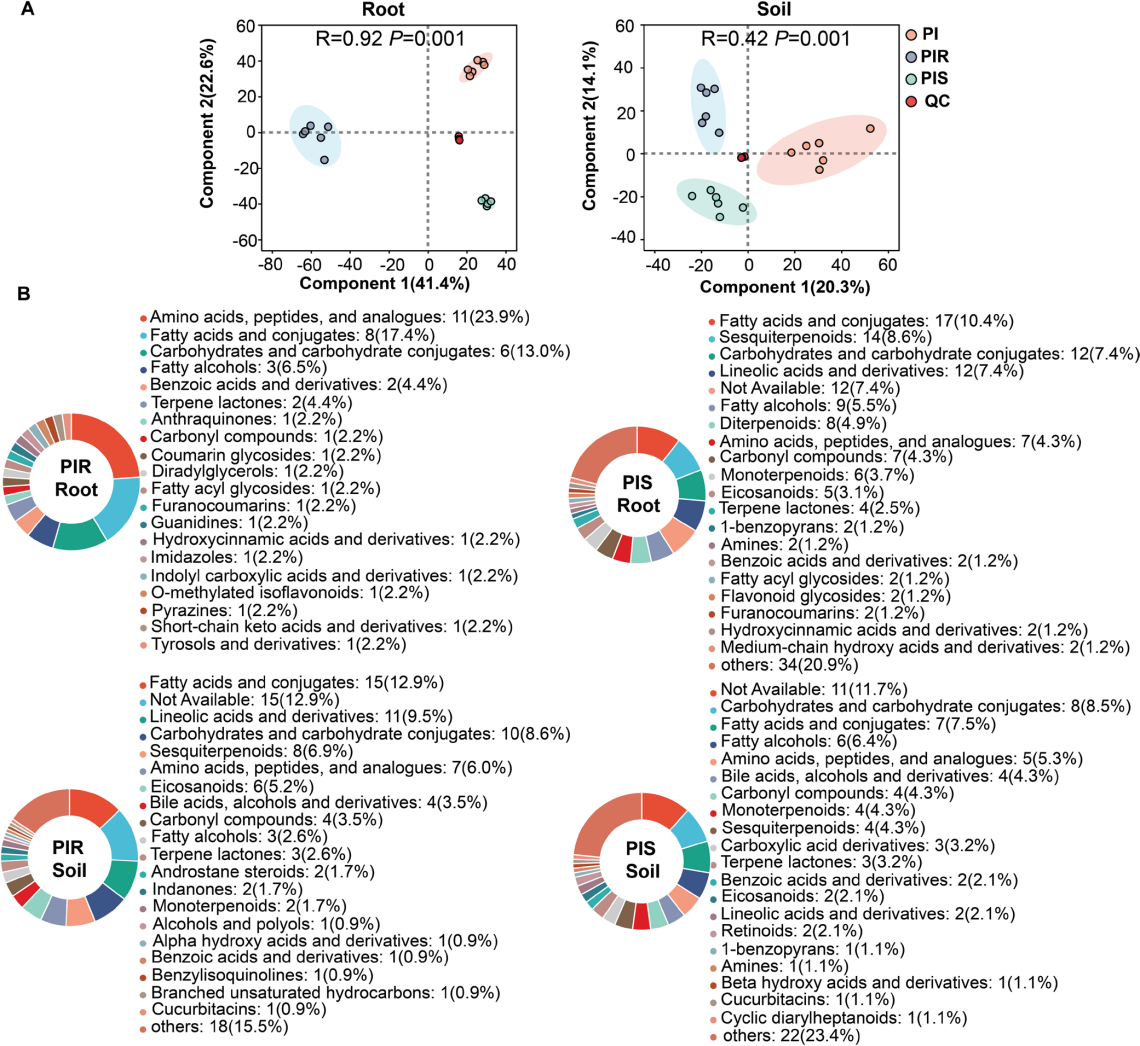
Metabolite changes in allelopathic rice root and rhizosheath soil under PI, PIR and PIS treatments. PI, PIR and PIS represent monocultured allelopathic rice with no barnyardgrass, allelopathic rice co‐cultured with quinclorac‐resistant and ‐susceptible barnyardgrass, respectively. (A) PLS‐DA (Partial Least Squares Discriminant Analysis) score plots displaying differences in the chemical composition of allelopathic rice root and rhizosheath soil metabolites under barnyardgrass stress. QC (Quality Control) represents the quality control measures implemented to ensure the reliability and accuracy of the data. Metabolite data were Pareto‐scaled before analysis. The ellipse represents the 95% confidence interval. Statistical significance of group separation was evaluated by ANOSIM (B) Pie charts based on counts of HMDB (Human Metabolome Database) taxonomy (subclass) for up‐regulated metabolites in allelopathic rice roots and rhizosheath soil.

A total of 9035 and 4554 metabolites in rice roots and rhizosheath soil samples were detected, respectively, using non‐targeted UPLC‐QTOF/MS. The PLS‐DA models indicated that the QC samples clustered together, and the accumulated metabolites among PI, PIR, and PIS were distinctly separated. More specifically, component 1 and component 2 explained 64% and 34.4% of the variance, respectively, indicating significant differences between the treatments for rice roots and attached rhizosheath soil (Figure 2A).

Based on the threshold for significantly different metabolites (VIP ≥ 1 and *p* < 0.05), types of DEMs were identified. Compared with the control, there were 46 and 163 significantly abundant metabolites in rice roots under PIR and PIS, respectively. Assigned to the HMDB database, these metabolites were matched and classified into 20 and 51 HMDB subclasses, respectively (Figure 2B). Among them, amino acids, peptides, and analogues (23.9%) were the dominant subclass in PIR. In contrast, terpenoids (sesquiterpenoids 8.6%, diterpenoids 4.9%, monoterpenoids 3.7%, and terpene lactone 2.5%) were the principal subclass in PIS.

In rice rhizosheath soil, 116 (in PIR) and 94 (in PIS) significantly enriched metabolites were classified into 38 and 42 HMDB subclasses (Figure 2B). Fatty acids and conjugates (12.9% in PIR, 7.5% in PIS), carbohydrates and carbohydrate conjugates (8.6% in PIR, 8.5% in PIS) were the main subclasses both in both PIR and PIS treatments. Moreover, terpenoids remained the predominant subclass in PIS, accounting for 11.9% (monoterpenoids 4.3%, sesquiterpenoids 4.3%, and terpene lactone 3.3%). Overall, terpenoids and amino acids were the key allelochemicals in rice to alleviate quinclorac‐susceptible and ‐resistant barnyardgrass, respectively.

To further identify specific compounds, Weighted Gene Co‐expression Network Analysis (WGCNA) was used to explore the correlation between key metabolites and physiological characteristic parameters (FPKM < 1). Highly correlated metabolite clusters in WGCNA were defined as different colour modules, with metabolites in the same cluster being highly correlated. Unselected metabolites were classified into grey modules. Six and eight co‐expression modules in rice roots and rhizosheath soil were identified, respectively (Figure S2). According to the correlation between modules and physiological indicators, the blue module was positively correlated with root SA and JA concentration, and leaf *Pn* value. The yellow module exhibited significant positive correlation with leaf thickness. A total of 176 metabolites belonged to the blue module, with most metabolites dominantly classified as terpenoids (sesquiterpenoids: 9.3%, diterpenoids: 5.2%, and monoterpenoids: 3.5%), fatty acids and conjugates subclass (10.4%) (Figure S3). The yellow module contained 73 metabolite compounds, predominantly classified as amino acids, peptides, and analogues (16.7%), fatty acids and conjugates (15.2%) (Figure S3). Interestingly, the blue module metabolites were dramatically increased in PIS, while the yellow module metabolites significantly increased in PIR (Figure S3).

In rhizosheath soil, 72 metabolites in the yellow module were mainly identified as fatty acids and conjugates (19.1%), carbohydrates and carbohydrate conjugates (10.9%) (Figure S3), which were positively correlated with *Pn*, SA and JA concentrations (Figure S2). Additionally, their abundance increased in both PIR and PIS treatments (Figure S3). Integrative analysis of transcriptomics and metabolomics found that amino acid‐associated nitrogen metabolism and terpenoid biosynthesis were significantly enriched in PIR and PIS, respectively (Figure S11). These findings further support that terpenoids are the primary allelochemicals in allelopathic rice responding to susceptible barnyardgrass stress, while amino acids are the dominant allelochemicals adapting to resistant barnyardgrass stress. Particularly, terpenoid accumulation has a positive correlation with root SA and JA signalling.

### 2.6 Differential Rhizosheath Microbiomes Are Enriched in Allelopathic Rice in Response to ‐Resistant and ‐Susceptible Barnyardgrass Stress

**FIGURE 3:**
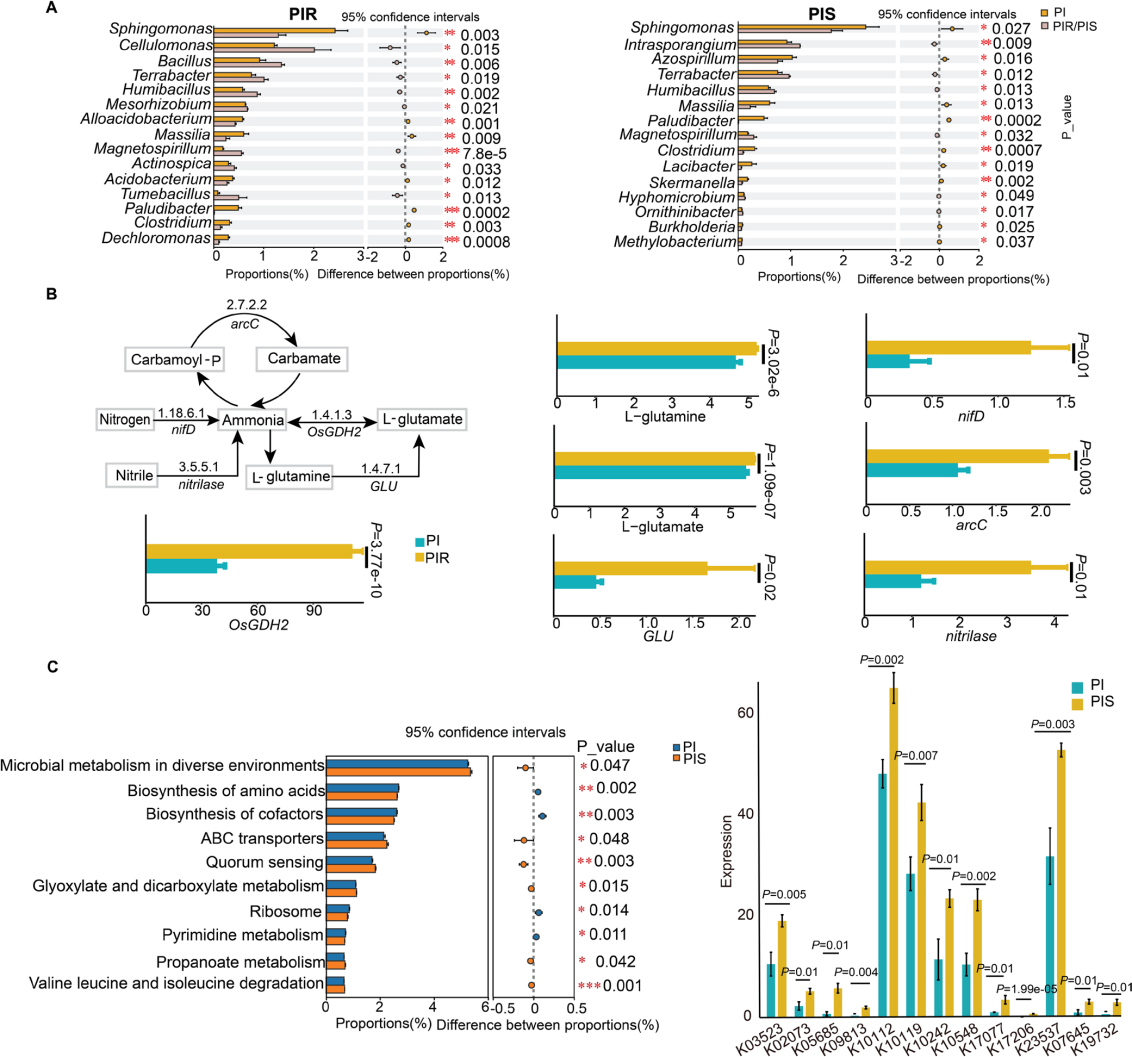
The composition and function of allelopathic rice rhizosphere microbiota affected by barnyardgrass. PIR and PIS represent allelopathic rice co‐cultured with quinclorac‐resistant and ‐susceptible barnyardgrass, respectively. (A) Differences in relative abundance of bacteria between PI and PIR/PIS treatments. The bar plot shows the mean proportions of differential bacteria. Genus‐level abundance was quantified using the RPKM method. (B) Up‐regulated nitrogen metabolism in allelopathic rice under the PIR treatment. L‐glutamine and L‐glutamate were up‐regulated metabolites, and *OsGDH2, the* genes in allelopathic rice roots; *nifD*, *arcC*, *GLU*, and nitrilase were up‐regulated genes and enzymes in bacteria. (C) Significantly different KEGG pathways between active bacteria in PI and PIS treatment (left panel). Significantly upregulated KEGG Orthology (KO) identifiers involved in the ABC transporter and quorum sensing pathways under the two treatments (right panel). The first ten KOs are associated with the ABC transporter pathway, and the last two belong to the quorum sensing pathway.

The impact of different barnyardgrass stresses on the community structure and microbial diversity of the rhizosheath microbiome in allelopathic rice was assessed through metagenomic sequencing. In our study, the majority of rhizosheath microbes in allelopathic rice were identified as bacteria (approximately 98%), while the remaining 2% comprised archaea, eukaryota, and viruses (Figure S4). PCoA models demonstrated a clear separation in the rhizosheath microbiome of allelopathic rice among PIR, PIS, and the control (Figure S5). According to the bacterial community composition analysis, 322 and 176 bacterial genera showed a significant (*p* < 0.05) increase in relative abundance in PIR and PIS, respectively (Table S3). The analysis of the top 15 genera showed that *Terrabacter*, *Humibacillus*, and *Magnetospirillum* were dramatically enriched bacterial genera in allelopathic rice under barnyardgrass stress, and their relative abundance increased by 34%, 51%, and 201%, respectively, in PIR, and by 29%, 20%, and 61%, respectively, in PIS (Figure 3A). Apart from the commonly increased genera, five genera (*Cellulomonas*, *Bacillus*, *Mesorhizobium*, *Actinospica*, and *Tumebacillus*) showed a significant (*p* < 0.05) increase (66%, 48%, 7%, 41%, and 501%, respectively) in PIR, while three genera (*Intrasporangium*, *Hyphomicrobium*, and *Ornithinibacter*) were significantly (*p* < 0.05) increased (27%, 18%, and 26%, respectively) in PIS.

Based on KEGG functional annotation analysis, *nifD* [nitrogenase molybdenum‐iron protein alpha chain (1.18.6.1)], *GLU* [glutamate synthase (ferredoxin) (1.4.7.1)], nitrilase (3.5.5.1), and *arcC* [carbamate kinase (2.7.2.2)] were involved in nitrogen metabolism, showing significant (*p* < 0.05) increase (64%, 185%, 82%, and 26%, respectively) in its relative abundance in the PIR treatment (Figure 3B). In the PIS treatment, the ABC transporter and quorum sensing pathways were significantly (*p* < 0.05) enriched (Figure 3C). Specifically, K10112, K10119, K10242, and K10548 (ABC transporter pathway) responsible for oligosaccharides transporter were dramatically (*p* < 0.05) increased by 35%, 50%, 107%, and 124%, respectively as well as K07645 and K19732 (quorum sensing pathway) were significantly (*p* < 0.05) upregulated by 256% and 448%, respectively (Figure 3C). Taxonomic attribution showed that ABC transporter‐related KOs (e.g., K05685, K09813, K17077) were mainly associated with *Bacillus*, *Caulobacter*, and *Cupriavidus*, while the quorum sensing‐related KO K07645 was linked to *Enterobacter*, *Hanamia*, and *Dyella* (Table S4).

To elucidate the correlations between root metabolites and core microbes in allelopathic rice rhizosheath soil under quinclorac‐resistant and ‐susceptible barnyardgrass stress, a co‐occurrence network analysis was conducted to analyse the correlation between specific metabolites and bacterial genera (Figure S6). Most correlations between metabolites and bacteria were positive in both PIR and PIS treatments. The prominent metabolites (L‐homoserine, L‐3‐cyanoalanine, and sarcosine) and the key bacteria (*Bacillus*, *Cellulomonas*, and *Actinospica*) were detected in the PIR network. Conversely, in the PIS network, carnosic acid, phytocassane B, and ipomeatetrahydrofuran were the main metabolites, with *Ramlibacter*, *Paludibacter*, and *Magnetospirillum* as the key taxa.

Further analysis considering environmental factors revealed that plant‐available soil nitrogen concentration was positively interacted with the enrichment of rhizosheath bacteria (*Cellulomonas*, *Humibacillus*, and *Terrabacter*; Figure S7). Several bacterial taxa associated with nitrogen metabolism were identified as core microbes, critical for the secretion of amino acids in rhizosheath soil networks under quinclorac‐resistant barnyardgrass infestation. Collectively, in allelopathic rice rhizosheath soil, distinct bacterial communities and functional gene pathways were enriched in response to ‐resistant and ‐susceptible barnyardgrass stress, respectively.

### 2.7 Combined Actions of Allelopathic Rice Amino Acids and Rhizosheath Beneficial Bacteria Contribute to an Increase in Plant‐Available Soil Nitrogen

Through random forest analysis, the importance of amino acids and bacteria on the plant‐available soil nitrogen was determined (Figure S8). Ten amino acids, N‐phenylacetylaspartic acid, sarcosine, pipecolic acid, L‐homoserine, L‐aspartic acid, L‐histidine, L‐norleucine, N‐acetyl‐L‐glutamate 5‐semialdehyde, L‐glutamic acid, and L‐3‐cyanoalanine were identified as key predictors of plant‐available soil nitrogen concentration (AN) based on their high variable importance scores in the model. Similarly, seven bacterial genera—*Cellulomonas*, *Bacillus*, *Terrabacter*, *Mesorhizobium*, *Humibacillus*, *Actinospica*, and *Magnetospirillum—also* exhibited high importance values, indicating their strong predictive power for AN.

To assess the relative contributions of biochemical and microbial factors to plant‐available nitrogen (AN), principal component analysis (PCA) was performed on the selected amino acids and bacterial genera. The first principal components—amino acid PC1 and bacteria PC1—explained 88.7% and 91.4% of the total variance, respectively (Figure S9).

**FIGURE 4:**
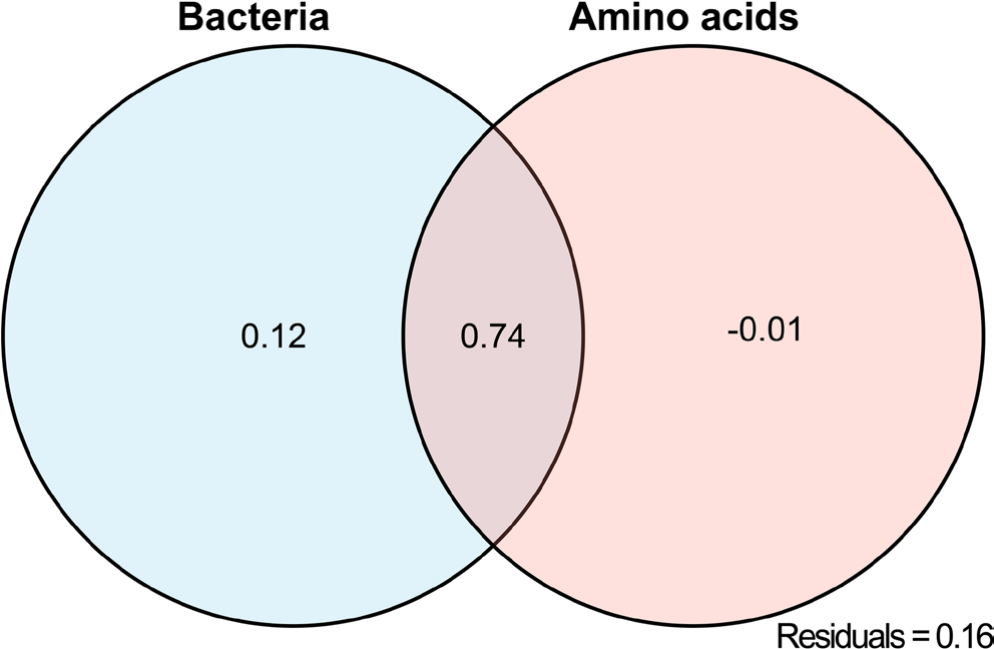
Variation partitioning analysis (VPA) showing the independent and shared contributions of bacterial communities and amino acids to the variation in plant‐available soil nitrogen under the PIR treatment. The diagram shows that bacterial communities uniquely explained 12% of the variance, amino acids explained −1% (adjusted *R* ^2^), and their shared contribution was 74%. Residual variance accounted for 16%. Negative adjusted *R* ^2^ values may result from limited sample size or variable collinearity and are interpreted as no unique contribution.

These two components were then used in a variation partitioning analysis (VPA) to quantify their unique and shared contributions to the variation in AN (Figure 4). The total explained variation was 84%, with 12% uniquely attributed to bacteria PC1, −1% to amino acid PC1, and 74% shared between them. The remaining 16% was unexplained. To assess robustness, a stratified bootstrap sensitivity analysis (10 000 resamples) was performed, confirming that the VPA results were not affected by the imputation procedure (Table S5). These results suggest that interactions between amino acids and bacterial communities are key drivers of soil nitrogen availability, with bacterial communities independently exerting significant influence on nitrogen dynamics.

### 2.8 Allelopathic Amino Acids Can Suppress Barnyardgrass Growth and Are Safe to Rice Seedlings

**FIGURE 5:**
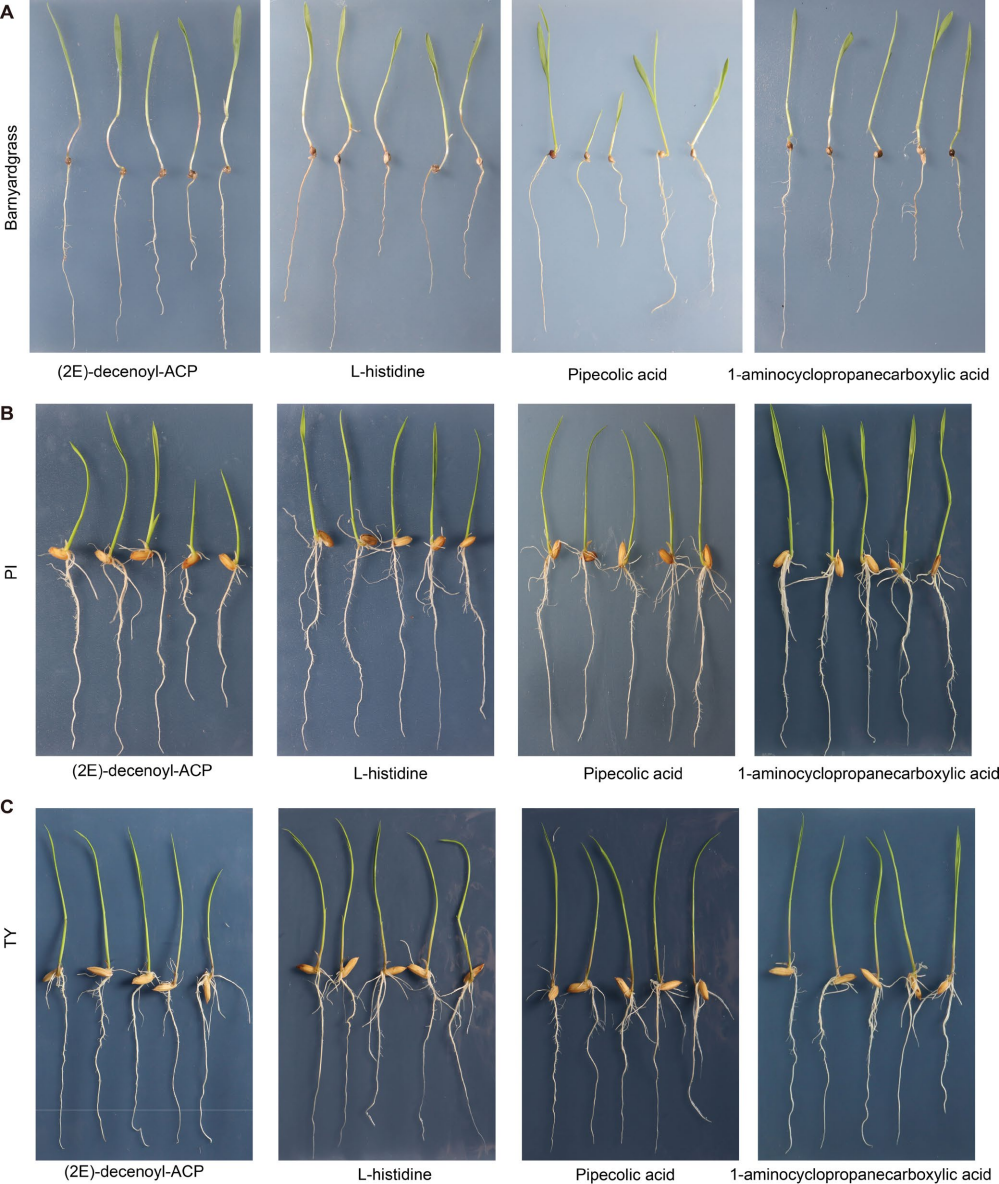
Effects of the four candidate amino acids on the seedling growth of barnyardgrass (A), allelopathic (PI312777, PI) (B) and non‐allelopathic (Tianyouhuazhan, TY) (C) rice. The first seedling on the left in each panel is the control (no amino acid treatment), and the remaining four seedlings, from left to right, were treated with the amino acid concentration of 40, 80, 120, and 160 μM, respectively.

**FIGURE 6:**
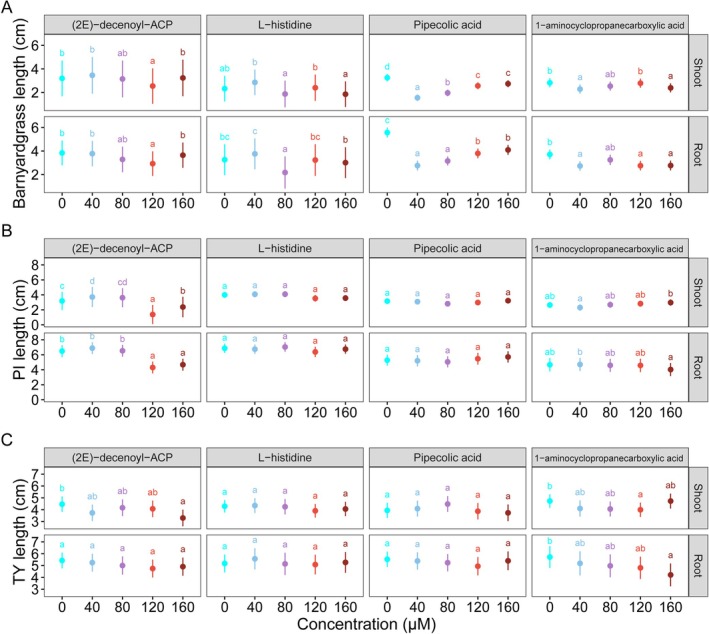
Effect of the amino acids ((2E)‐decenoyl‐ACP, L‐histidine, pipecolic acid, and 1‐aminocyclopropanecarboxylic acid) on the seedling growth of barnyardgrass (A), allelopathic (PI312777, PI) (B) and non‐allelopathic (Tianyouhuazhan, TY) (C) rice. Data are shown as means ±95% confidence intervals using linear mixed‐effects models, with individual dish included as a random effect (*n* = 3). Different letters indicate significant differences among concentration treatments, as determined by Tukey's HSD tests based on linear mixed‐effects models.

Amino acids with significantly increased abundance in allelopathic rice were used to assess their potential role in suppressing barnyardgrass growth (Table S6). Results showed that four amino acids ((2E)‐decenoyl‐ACP (traditional name: cycloleucine), L‐histidine, pipecolic acid, and 1‐aminocyclopropanecarboxylic acid) were equally effective in inhibiting barnyardgrass seedling growth (Figures 5 and 6).

(2E)‐decenoyl‐ACP inhibited barnyardgrass shoot and root growth by 20% (0.65 cm) and 24% (0.91 cm; *p* < 0.01) at 120 μM, L‐histidine significantly inhibited root length by 33% (1.08 cm) at 80 μM (*p* < 0.001). Pipecolic acid exhibited similar inhibitory effects on both root and shoot length at all concentrations tested, with the stronger inhibition at lower concentrations (40 and 80 μM). Specifically, shoot length was reduced by 52% (1.71 cm) and 40% (1.29 cm) at 40 and 80 μM, respectively, while root length decreased by 50% (2.81 cm) and 43% (2.42 cm; *p* < 0.001). 1‐aminocyclopropanecarboxylic acid significantly reduced barnyardgrass shoot and root length by 19% (0.54 cm) and 26% (0.98 cm) at 40 μM (*p* < 0.001), and 15% (0.43 cm) and 25% (0.94 cm) at 160 μM (*p* < 0.001), respectively.

The effect of these four amino acids on rice seedling growth was also tested. Results showed that L‐histidine and pipecolic acid at all tested concentrations had no inhibitory effect on allelopathic (PI) and non‐allelopathic (TY) rice seedling growth (Figures 5 and 6). Notably, (2E)‐decenoyl‐ACP at 40 μM increased the PI shoot length by 16% (0.52 cm). However, at higher concentrations (120 and 160 μM), (2E)‐decenoyl‐ACP inhibited the PI shoot and root elongation, and the TY shoot length was also suppressed at 160 μM. 1‐aminocyclopropanecarboxylic acid had no negative effects on PI growth, but significantly (*p* < 0.01) inhibited the TY shoot length at 120 μM by 16% (0.74 cm), and root length at 160 μM by 26% (1.51 cm). Taken together, the results suggested that the selected amino acids are inhibitory for barnyardgrass seedling growth at the concentrations tested, and two amino acids, pipecolic acid and L‐histidine, showed no adverse effects on allelopathic and non‐allelopathic rice seedlings.

**FIGURE 7:**
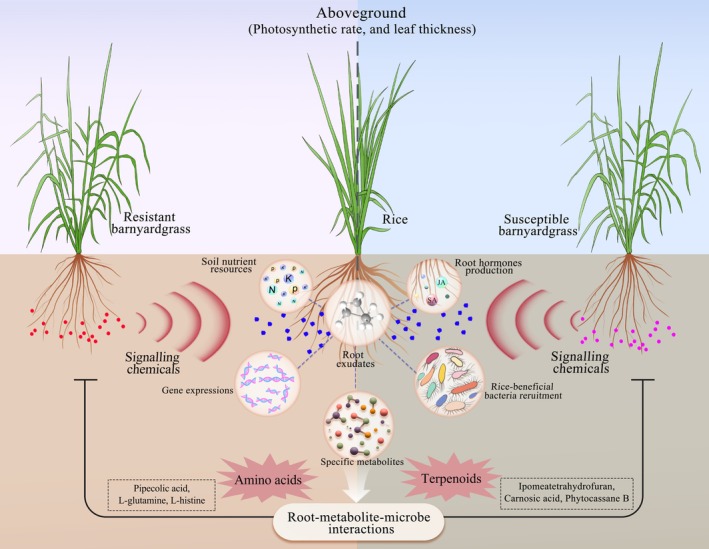
Schematic drawing illustrating allelopathic rice secretes distinct metabolites to modulate rhizosheath microbiota to improve allelopathic rice tolerance against quinclorac‐resistant and ‐susceptible barnyardgrass.

In summary, our results reveal that under quinclorac‐susceptible and ‐resistant stress conditions, allelopathic rice exudate distinct metabolites (e.g., terpenoids, amino acids) and affect the rhizosheath microbiota, which in turn promotes plant growth under barnyardgrass stress (Figure 7).

## 3 Discussion

This study characterised the physiology, gene expression, metabolite composition and rhizosheath soil microorganisms of allelopathic rice under quinclorac‐susceptible and ‐resistant barnyardgrass stress. Consistent with previous studies, terpenoids are the major allelochemicals in allelopathic rice responding to quinclorac‐susceptible barnyardgrass stress, and their levels are positively correlated with root salicylic acid (SA) and jasmonic acid (JA) signalling pathways. Moreover, terpenoid compounds recruited beneficial bacteria with functional gene pathways (such as quorum sensing and oligosaccharides transporter) to enhance weed suppression. In contrast, amino acid‐enriched allelochemicals were identified as key allelochemicals in allelopathic rice under quinclorac‐resistant barnyardgrass stress. In addition, these allelochemicals promoted nitrogen‐fixing bacteria enrichment, leading to increased plant‐available soil nitrogen. Our study provides a novel complex plant‐metabolite‐microbe defence network in allelopathic rice against barnyardgrass stress, offering valuable insights for developing sustainable weed control and management strategies in paddy fields. However, why allelopathic rice responds differently to quinclorac‐susceptible (S) and ‐resistant (R) barnyardgrass is unknown (Peng et al. 2019). Quinclorac is an auxinic herbicide whose mode of action has been suggested to involve perturbation of auxin homeostasis and induction of ethylene biosynthesis (Grossmann 2010; Grossmann and Kwiatkowski 1993, 2000). Indeed, quinclorac resistance in the R barnyardgrass very likely involves auxin‐ethylene crosstalk (Peng et al. 2019), although the exact mechanisms and molecular basis of quinclorac resistance is yet to be determined. In this current study, our transcriptomic analysis revealed differential expression of auxin‐ and ethylene‐related genes in allelopathic rice under stress from R versus S barnyardgrass (Table S7), suggesting that hormone crosstalk may contribute to the contrasting rice responses to R and S barnyardgrass and warrants further investigation. Notably, we did observe early flowering and higher photosynthesis rates in the R than the S lines (Figure S1; Li, Wang, et al. 2020), highlighting intrinsic physiological differences between R and S barnyardgrass that may shape rice responses. Moreover, only the S and R barnyardgrass lines (for better control of the genetic background) were used in the study, and if the observed results are applicable to other S and R barnyardgrass populations as well as populations resistant to other commonly used herbicides in paddy fields is worth further investigations.

### 3.1 Distinct Allelochemicals in Mediating Allelopathic Rice Response to Quinclorac‐Susceptible and Resistant Barnyardgrass

Consistent with previous findings that terpenoids are the main allelochemicals for rice against barnyardgrass (Kato‐Noguchi et al. 2010; Kato‐Noguchi and Ino 2003; Li, Zhao, and Kong 2020), our study also observed that terpenoids are primary compounds in quinclorac‐susceptible barnyardgrass‐co‐cultured rice. Up‐regulation of genes in momilactone A biosynthesis in PIS treatment, including *OsCPS4* (*Os04g0178300*), and *CYP99A1* (*Os04g0178400*), are also similar to previous studies (Sultana et al. 2023). In contrast, both genes were downregulated in the PIR treatment, suggesting that terpenoid‐based allelopathic pathways maybe not activated under the stress from quinclorac‐resistant barnyardgrass. Furthermore, our study found a positive correlation between SA, JA concentration and terpenoids accumulation in allelopathic rice roots (Figure S2). Expression levels of the two key genes, *PR1* (associated with SA signalling) and *AOS* (associated with JA signalling), were activated by quinclorac‐susceptible barnyardgrass stress (Figure 1D). Both SA and JA are known to induce the synthesis of terpenoids in plants, thereby enhancing plant defence against pests and pathogens (Kerchev et al. 2012; Kiyama et al. 2021). In addition to activating defence‐related secondary metabolism, the elevated root SA and JA levels observed under the PIS treatment are indicative of a defence‐priming state, enabling rapid response to barnyardgrass stress with limited metabolic costs (Conrath et al. 2015; Mauch‐Mani et al. 2017). Consistent with this notion, allelopathic rice under PIS maintained higher *Pn* and lower *Cᵢ*, suggesting improved photosynthetic efficiency and a coordinated reallocation of carbon resources that supports both defence activation and growth. Taken together, these coordinated hormonal, metabolic, and physiological responses highlight terpenoid biosynthesis as a primary allelopathic strategy employed by rice in response to quinclorac‐susceptible barnyardgrass.

In contrast, in quinclorac‐resistant barnyardgrass cocultured rice, the terpenoid pool had little changes. However, amino acids, particularly protein amino acids (PAAs, L‐histidine, and L‐glutamate) and non‐protein amino acids (NPAAs, (2E)‐decenoyl‐ACP, and pipecolic acid), were significantly increased in the allelopathic rice root tissue (Figure S3). PAAs have been identified as allelochemicals involved in plant defence responses (Shiade et al. 2024). In addition to their primary function in protein synthesis, protein amino acids are known to contribute significantly to stress responses (Heinemann and Hildebrandt 2021; Hildebrandt et al. 2015). For instance, histidine has been shown to effectively inhibit seed germination and tubercle formation in holoparasitic broomrape ( *Orobanche ramosa* ) (Vurro et al. 2006). Our study also demonstrated PAAs (L‐histidine) potential effects in suppression of barnyardgrass seedling growth (Figures 5 and 6).

In parallel, non‐protein amino acids (NPAAs) exhibit selective herbicidal activity through molecular mimicry. These compounds often act as structural analogs of protein amino acids, competitively disrupting protein synthesis. For instance, L‐3,4‐dihydroxyphenylalanine selectively inhibits weeds (e.g., *Sinapis arvensis* ) without affecting cereal crops (Soares et al. 2014), while mimosine targets broadleaf species through germination suppression (Xuan et al. 2013). Our study identified three NPAAs (2E)‐decenoyl‐ACP, 1‐aminocyclopropanecarboxylic acid, and pipecolic acid, with pipecolic acid exhibiting particularly potent activity, inhibiting 50% of barnyardgrass shoot and root growth at 40 μM, but not much so at the higher concentrations (Figures 5 and 6). This observed growth suppression may result from the misincorporation of NPAAs into nascent polypeptides due to structural similarity, leading to the production of non‐functional proteins (Thives Santos et al. 2024). At elevated concentrations, pipecolic acid transport via its cognate amino acid carriers becomes saturated, limiting further intracellular uptake (Dinkeloo et al. 2018), while simultaneously triggering detoxification processes, such as glutathione conjugation (Coleman et al. 1997) and vacuolar sequestration (Singh et al. 2016), that may collectively diminish its inhibitory effects. However, because our non‐targeted LC–MS/MS approach only provided relative abundances without quantifying in situ soil NPAA concentrations, the actual rhizosphere NPAA levels remain to be determined to justify their growth inhibition effects at physiologically relevant concentrations.

Although terpenoids are currently considered the principal allelochemicals involved in rice‐mediated suppression of barnyardgrass (Kato‐Noguchi 2011; Sultana et al. 2023), with well‐documented roles in direct growth inhibition (Kato‐Noguchi and Ino 2013), our study is the first to identify amino acids as potential allelopathic agents. However, it remains unclear whether these amino acids represent a broad‐spectrum defence strategy specifically induced in response to quinclorac‐resistant barnyardgrass. Future research using barnyardgrass biotypes resistant to herbicides of different modes of action (e.g., penoxsulam and cyhalofop‐butyl) deserves investigation.

### 3.2 Terpenoid and Amino Acid Metabolites Likely Modulate the Structure of Weed‐Suppressive Rhizosheath Microbiomes in Allelopathic Rice

Several recent studies have revealed that plants recruit beneficial bacteria to enhance plant stress tolerance and promote plant growth when exposed to biotic stress (Carrión et al. 2019; Gulati et al. 2020; Jousset et al. 2011; Lendzemo et al. 2007). Consistent with previous findings that allelochemicals more strongly affect soil bacteria than fungi (Lorenzo et al. 2013), our study also observed that approximately 98% of the microbial genera in the allelopathic rice rhizosheath soil were bacteria (Figure S4). PCoA models demonstrated a clear separation in the rice rhizosheath microbiome between PIR, PIS, and the control (PI) treatment (Figure S5), indicating that the suppressive effect of allelopathic rice on barnyardgrass growth occurs likely via the dynamic modulation of distinct rhizosheath soil microbial communities. In our study, *Cellulomonas* and *Intrasporangium* were significantly enriched bacterial genera under ‐resistant and ‐susceptible barnyardgrass stress, respectively (Figure 3). Further analysis showed that amino acid enrichment may affect the abundance of bacteria, and bacterial communities exerted a significant independent contribution to nitrogen availability. Notably, their interactions accounted for the majority of the variation in plant‐available soil nitrogen (AN) under the resistant barnyardgrass stress (Figure 4). To clarify the contribution of rhizosheath microbiomes to allelopathic effects in growth and nutrient uptake enhancement in allelopathic rice, sterilised soil could be used as controls for future studies.

Root exudates comprise a wide range of compounds, including organic acids, amino acids, sugars, and terpenoids, mediating interactions between plants and rhizobacteria (Badri and Vivanco 2009). Recent studies show that secondary metabolites can shape microbiome composition, and these metabolites include glucosinolates (Chroston et al. 2024), flavonoids (Yu et al. 2021), coumarins (Voges et al. 2019), benzoxazinoids (Cotton et al. 2019), and triterpenes (Huang et al. 2019). In our study, terpenoids (e.g., sesquiterpenoids, monoterpenoids, diterpenoids, and terpene lactone) were identified to be the main allelochemicals in susceptible barnyardgrass‐cocultured allelopathic rice. Notably, a previous study showed that triterpenoids mediated the assembly of 52% of *Arabidopsis thaliana* ‐specific root microbiomes (Huang et al. 2019). Diterpenoids and terpenes also influence rhizosheath bacterial communities (Chou et al. 2023; Murphy et al. 2021). Our study revealed that diterpenoids had positive correlation with *Ramlibacter*, *Paludibacter*, and *Magnetospirillum*. *Ramlibacter*, and *Magnetospirillum* are well known plant‐beneficial bacteria because they can promote plant growth (Diao et al. 2024; Shirsat and Suthindhiran 2025). Some pathways significantly enriched in PIS treatment were important to soil health and plant growth. For example, the quorum sensing (QS) pathway enriched in PIS, is the key pathway of microbe communication (Kostylev et al. 2019; Quiñones et al. 2005), and these QS signal deficient microbes can hardly colonise the host plants (Bauer and Mathesius 2004; Quiñones et al. 2005). The QS also has plant growth promotion effects, which have been observed in rice (Steindler et al. 2009). The K07645 and K19732, members of the quorum sensing pathway, were also significantly enriched in PIS (Figure 3C). Moreover, oligosaccharide transporters (ABC transporters) were also significantly enriched in PIS. Oligosaccharides are soluble, readily available to microorganisms, and rapidly utilised for essential cellular functions such as respiration, anabolism, growth, and carbon storage (Gunina and Kuzyakov 2015). We hypothesize that under susceptible barnyardgrass stress, allelopathic rice plants enrich beneficial bacteria via terpenoid accumulation. These bacteria enhance their ability to capture the limited available sugars by upregulating ABC transporters and promote the colonisation of beneficial bacteria in the rhizosphere by upregulating QS, thereby helping rice ameliorate barnyardgrass stress. Further studies with the bacterial isolates are needed to test this hypothesis.

### 3.3 Amino Acid Secretion by Allelopathic Rice Roots Promotes N Uptake and Enhance Tolerance to Quinclorac‐Resistant Barnyardgrass Stress

Amino acids shape plant‐microbe interaction, through providing signalling molecules, defence compounds, and nutrients (Moormann et al. 2022). More beneficial bacteria were enriched in resistant barnyardgrass‐co‐cultured rice root system, including nitrogen‐fixing, ammonifying bacteria, and cellulolytic bacteria (Figure 3A). These bacteria participate in nitrogen metabolism, and their interactions in the nitrogen cycle help maintain the dynamic balance of nitrogen in the ecosystem and prevent nitrogen loss. In our study, the concentrations of L‐homoserine, L‐histidine, L‐3‐cyanoalanine and sarcosine were positively correlated with the abundance of nitrogen‐fixing (e.g., *Mesorhizobium*, *Paenibacillus*, *Nocardia* and *Tumebacillus*), ammonifying (*Bacillus*) and cellulolytic bacteria (*Cellulomonas*; Figure S6). The observed positive correlations between specific amino acids and functional microbial taxa suggest a tightly coordinated nitrogen metabolism network. The combined use of L‐methionine and *Rhizobium* increased the yield of chickpeas and nitrogen concentration in the grains (Rafique et al. 2022). Our study also revealed that the plant‐available soil nitrogen was significantly increased in resistant barnyardgrass‐co‐cultured allelopathic rice rhizosheath soil (Table 2). The observed increase in total nitrogen concentration was attributed to an upregulation of biological nitrogen fixation, a crucial metabolic process exclusively by prokaryotes (Coale et al. 2024). Notably, co‐culture of allelopathic rice with resistant barnyardgrass significantly promoted N fixation in the rice rhizosheath soil, via the upregulation of key N‐fixation gene (*nifD*), which also positively corelated with high abundances of *Mesorhizobium* and *Cellulomonas*, indicating a unique interaction between the diazotrophs.

The co‐enrichment of the cellulolytic bacterium *Cellulomonas*, the N fixation gene *nifD*, and downstream nitrogen metabolism‐related genes and enzyme (*arcC*, nitrilase, and *GOGAT*) points to the establishment of a coordinated nitrogen‐processing network in the rice rhizosheath facilitated by cellulose degradation under quinclorac‐resistant barnyardgrass stress. Mechanistically, cellulose degradation by *Cellulomonas* may provide readily available carbon substrates to meet the high energy demand of diazotrophy (Leschine 1995; Schellenberger et al. 2012), while increased oxygen consumption creates localised microaerobic niches favourable for nitrogenase activity (Young et al. 2012). In this context, the upregulation of *nifD* reflects enhanced conversion of atmospheric N_2_ to ammonium. The increased ammonium flux generated by enhanced diazotrophy necessitates coordinated downstream nitrogen processing and assimilation within the rhizosheath microbial community. In this downstream processing step, the *arcC* gene, encoding carbamate kinase, converts carbamoyl phosphate and ADP into ammonia, carbon dioxide and ATP (Hennessy et al. 2018), thereby contributing both nitrogen and energy to microbial metabolism. In parallel, nitrilases, which hydrolyze nitriles to carboxylic acids and ammonia (Howden and Preston 2009), were enriched in relative abundance (Figure 3B), consistent with the increased availability of plant‐derived nitriles observed in this study (Figure S10). Finally, *GOGAT* converts ammonia into glutamate, preventing toxic ammonia accumulation and facilitating the assimilation and transfer of organic nitrogen within the rhizosheath (Senthilkumar et al. 2021). Collectively, the coregulation of *nifD*, *arcC*, *GOGAT*, and nitrilases contributes to the stabilisation of rhizosheath nitrogen cycling, functionally associated with the enrichment of *Cellulomonas*.

Following microbially‐mediated biological nitrogen fixation, plants assimilate inorganic nitrogen through specific transporters. Within plant tissues, ammonium ions are primarily incorporated into organic compounds via the glutamine synthetase/glutamate synthase pathway (Zayed et al. 2023). This enzymatic system catalyses the sequential synthesis of glutamine and glutamate, the foundational amino acids in plant nitrogen metabolism (Paschalidis et al. 2019). As the initial organic nitrogen carriers, glutamine and glutamate facilitate nitrogen remobilization for synthesising diverse nitrogenous compounds, ensuring dynamic nitrogen homeostasis in plants (Paschalidis et al. 2019; Zayed et al. 2023). In the PIR treatment, metabolomic analyses revealed a significant increase in the abundance of L‐glutamine and L‐glutamate in allelopathic rice roots, which is consistent with the up‐regulation of *OsGDH2* (*gdhA*) (Figure 3B). As a key enzyme involved in ammonium‐glutamate interconversion, elevated *OsGDH2* expression may support enhanced glutamate‐associated nitrogen metabolism and facilitate interactions between plant nitrogen assimilation and diazotrophic microorganisms in the rhizosheath. Our findings support a mechanistic model that the allelopathic rice interacts with quinclorac‐resistant barnyardgrass by alteration of the composition of root exudates, particularly by enhancing the release of amino acid signals. This alteration of exudate composition facilitates the recruitment of key diazotrophs, including *Azorhizobium* and *Burkholderia*, which enhances biological N fixation in the rhizosphere, thereby increasing plant‐available soil nitrogen and promoting plant N acquisition.

When exposed to resistant‐barnyardgrass stress, allelopathic rice established a self‐reinforcing nitrogen cycle through coordinated microbial partnership. However, this functional synergy was not orchestrated when exposed to susceptible barnyardgrass treatment. Although some nitrogen‐fixing bacteria were present in susceptible barnyardgrass co‐cultured rhizosheath, the plant‐available soil nitrogen showed no significant increase in our study (Tables 2 and S3). The discrepancy may be attributed to the absence of key cellulolytic taxa, such as *Cellulomonas*, which are required to support the coordinated nitrogen‐processing network observed under resistant‐barnyardgrass stress. In the absence of this functional support, nitrogen fixation in the susceptible‐barnyardgrass rhizosheath appears insufficient to translate into increased plant‐available soil nitrogen. Further studies are needed to unravel how cellulolytic taxa interact with other bacteria to regulate the nitrogen cycle.

Despite these insights, our study did not isolate and functionally validate specific microbial strains enriched under different treatments, which limits direct causal inference between individual taxa and their proposed ecological functions (e.g., nitrogen fixation, quorum sensing, and ABC transporters). Nevertheless, the integration of metabolomic, transcriptomic, and microbiome functional analyses provides compelling correlative evidence for plant‐mediated microbial recruitment. Future studies incorporating gnotobiotic systems, microbial synthetic communities, or strain‐level functional assays are essential to experimentally verify the roles of key microbial players and their interaction with specific root exudates. These approaches will help further elucidate the mechanistic underpinning rhizosphere functional shifts under biotic stress. In summary, this study represents the first comprehensive investigation into the specific defence mechanisms of allelopathic rice against quinclorac‐susceptible and ‐resistant barnyardgrass using multi‐omics approaches. Our study revealed differential interactions between allelopathic rice and quinclorac‐susceptible and ‐resistant barnyardgrass. On one hand, amino acids secreted by allelopathic rice roots not only inhibit barnyardgrass growth but also synergize with nitrogen‐metabolising bacteria to enhance nitrogen acquisition, thereby improving allelopathic rice tolerance to quinclorac‐resistant barnyardgrass. On the other hand, SA and JA signalling may contribute to a defence‐priming state, promoting terpenoid production and the recruitment of beneficial bacteria via functional gene pathways (e.g., ABC transporters and quorum sensing), ultimately suppressing quinclorac‐susceptible barnyardgrass growth. This study provides valuable insights into allelopathic interactions between rice and its major competitor weed species, with great potential for effectively managing both resistant and susceptible barnyardgrass.

## 4 Materials and Methods

### 4.1 Plant Materials and Experimental Design

A well‐known allelopathic rice cultivar ‘PI312777’ (PI) (Dilday et al. 2001) was used for this study. As the composition and concentration of signalling molecules are influenced by neighbouring plant species or biotypes (Kong et al. 2018; Yang et al. 2018), quinclorac‐resistant (R) and ‐susceptible (S) barnyardgrass lines were used. The R and S lines were characterised based on their dose‐responses to quinclorac, and resistance was confirmed by the survival rate analysis (Figure S12), following the procedures described in Yang et al. (2021). Both the R and S lines were derived from the progeny of a single R plant segregating for resistance traits, thereby minimising the background genetic variation. Rice and barnyardgrass seeds were surface sterilised (75% ethanol for 30 s, and 2.5% sodium hypochlorite for 3 × 15 min), and sown separately onto wet filter paper in 9‐cm diameter petri dishes, and maintained in a growth chamber at 28°C.

The experimental design included two treatment groups: PIR and PIS, where PI was co‐cultured with R or S barnyardgrass, respectively. PI monoculture without barnyardgrass was used as a control. Two uniform rice seedlings at the one‐leaf stage were transplanted into plastic pots (46 cm length × 35 cm width × 17 cm height), each containing 15 kg of dry paddy soil from rice fields. The air‐dried soil was sieved through a 2‐mm mesh to remove coarse material and plant residues. The soil contained 70 mg kg^−1^ nitrogen, 30 mg kg^−1^ phosphorus, and 170 mg kg^−1^ potassium, which falls within the suitable range for early‐stage rice growth. At the two‐leaf stage of rice, two one‐leaf‐stage barnyardgrass seedlings were transplanted into each plastic pot, with a co‐planting density ratio of 1:1 (rice:barnyardgrass). The distance between the transplanted barnyardgrass and rice was approximately 5 cm. Other weeds were manually removed to prevent additional competition. The experiment was conducted in a completely randomised design, with six independent biological replicates per treatment (pot = replicate, *n* = 6). Pots were randomly positioned at the start of the experiment and periodically rotated during the experimental period (April–June 2024) to minimise potential positional effects under outdoor conditions. Plants were grown outdoors under natural environmental conditions. Daily mean air temperature ranged from 13.7°C to 30.6°C, with monthly mean temperatures of 19.9°C (April), 23.2°C (May), and 21.3°C (June). Daily sunshine duration ranged from 0 to 12.0 h, reflecting variable cloud and rainfall conditions, and monthly precipitation totalled 346.1, 122.1, and 640.3 mm in April, May, and June, respectively. Pots were irrigated every 1–2 days, or as required. Soil moisture was monitored daily and maintained under moist to shallow‐flooded conditions (target water layer: approximately 0–2 cm). No additional fertiliser was applied during the experimental period.

### 4.2 Experimental Sample Collection

At the six‐leaf stage, physiological indices of the allelopathic rice were recorded. Unless otherwise stated, one rice plant was sampled from each pot, and each pot was treated as one independent biological replicate (*n* = 6). Leaf and root samples were collected from treated (co‐culture) and control (monoculture) rice plants on a pot basis. The same rice plant samples were used for transcriptomic and metabolomic analyses and were immediately frozen in liquid nitrogen and stored at −80°C until further processing. Rhizosheath soil samples, defined as the cohesive soil layer tightly adhering to the rice root surface upon excavation (Pang et al. 2017), were collected following a previously described method (Simmons et al. 2018), with minor modifications. Rhizosheath soil samples were immediately frozen at −80°C and used for metagenomic analyses. In addition, soil samples were collected from each pot for determination of soil physicochemical properties. These soil samples were air‐dried, sieved (2 mm), and stored at room temperature prior to analysis.

#### 4.2.1 Photosynthetic Rate and Leaf Morphological Parameters

At the six‐leaf stage, the youngest fully expanded leaves were randomly selected from each treatment group to determine the photosynthetic rate (*Pn*), stomatal conductance (*gs*), intercellular CO_2_ concentration (*C*_*i*_), and transpiration rate (*Tr*) using a portable photosynthesis system (LI‐6800, Li‐Cor, Lincoln, Nebraska, USA) from 9:00 to 11:30 AM on clear days. During measurements, leaves were exposed to a saturated photosynthetic photon flux density of 1600 μmol m^−2^ s^−1^ provided by red/blue light‐emitting diodes. The leaf chamber temperature was maintained at approximately 30°C, and the relative humidity was kept about 70%. The reference CO_2_ concentration was maintained at around 400 μmol mol^−1^, and the mean leaf‐to‐air vapour pressure difference was held at approximately 1.1 kPa. Flag leaf thickness was measured as the average thickness of the middle part of the leaf blade using a micrometre. One rice plant was measured from each pot, resulting in six biological replicates per treatment (*n* = 6).

#### 4.2.2 Extraction, Purification, and Determination of Phytohormones

The analysis of salicylic acid (SA) and jasmonic acid (JA) in rice leaves and roots was conducted following a previously described method (Yan et al. 2023). Approximately 200 mg of fresh plant material was subjected to cryogenic grinding using liquid N_2_, then extracted with 1 mL of 80% (v/v) HPLC‐grade methanol (Merck, Darmstadt, Germany) at 4°C overnight. The samples were centrifuged at 15 000 ***g*** for 10 min, and the residue was re‐extracted with 0.5 mL of 80% (v/v) methanol. The combined supernatants were vacuum freeze‐dried at −60°C and then reconstituted in 200 mL of 0.1 M sodium phosphate buffer (pH 7.8). The aqueous phase was purified using a pre‐washed Waters Sep‐Pak C_18_ cartridge (Waters, Milford, MA, USA) and eluted with 1.4 mL of 80% (v/v) methanol. The methanol eluate was vacuum freeze‐dried, and the resulting dried extract was dissolved in 40 μL of 10% (v/v) methanol for LC–MS/MS analysis using a Shimadzu LC‐20AD‐8030 Plus MS system (Shimadzu, Kyoto, Japan). SA and JA quantification was performed using samples from three independent pots per treatment, with one rice plant sampled per pot (*n* = 3).

#### 4.2.3 Determination of Samples Physicochemical Properties

Soil pH was measured using a pH electrode (Leici, Shanghai, China) in a solution at a soil:water ratio of 1:2.5. Prior to analysis, soil samples were air‐dried and sieved through a 2.0‐mm mesh. Total organic matter (TOM) was determined using the potassium dichromate volumetric method (Nelson and Sommers 1996). Alkali‐hydrolyzed nitrogen (AN), available phosphorus (AP), and available potassium (AK) were analysed using the diffusion method (Bremner 1965), the Olsen method (Olsen 1954), and the ammonium acetate extraction flame photometry method (Helmke and Sparks 1996), respectively. Soil cation exchange capacity (CEC) was determined using the BaCl_2_‐MgSO_4_ method (Rhoades 1982). Soil electrical conductivity (EC) was measured using the electrode method (DDSJ‐308A, Leici, Shanghai, China), in accordance with the national standard. AN was measured using soil samples collected from six pots per treatment (*n* = 6), whereas other soil physicochemical properties (pH, TOM, AP, AK, CEC, and EC) were determined using samples from three pots per treatment (*n* = 3). Additionally, the total nitrogen content in rice plants was determined using the Kjeldahl digestion method (Isaac and Johnson 1976). One rice plant harvested from each pot was used as an independent biological replicate and six replicates were used (*n* = 6).

### 4.3 Multi‐Omics Analyses

#### 4.3.1 Multi‐Omics Were Used in This Study and Each Was Described as Below

The transcriptome analysis (RNA‐seq) was undertaken using the Novaseq 6000 (Illumina), to identify changes in gene expression in the allelopathic rice root tissue in the presence and absence of quinclorac‐susceptible and ‐resistant barnyardgrass. At the six‐leaf stage, rice root samples were collected from one rice plant per pot, with six independent pots per treatment serving as biological replicates (*n* = 6). Approximately 0.5 g of frozen root samples were used. RNA from rice root samples was extracted using TRIzol Reagent (Invitrogen, Carlsbad, California, USA), purified using Plant RNA Purification Reagent (Invitrogen, Carlsbad, California, USA), and sequenced using the Illumina NovaSeq 6000 platform by Shanghai Majorbio Bio‐pharm Technology Co. Ltd., China.

Raw paired‐end reads were trimmed and quality‐controlled using fastp (Chen et al. 2018). Clean reads were aligned to the rice reference genome using HISAT2 (Kim et al. 2015), and assembled using StringTie (Pertea et al. 2015). Gene expression levels were quantified using RSEM (Li and Dewey 2011) and normalised to TPM (transcripts per million). Across all samples (3 treatments: PIR, PIS, and PI, *n* = 18), RNA sequencing generated an average of 46.3 ± 4.6 (mean ± SD) million reads per sample. On average, 92.40% ± 0.22% of reads were uniquely mapped to the *Oryza sativa* reference genome IRGSP‐1.0, obtained from Ensembl Plants (http://plants.ensembl.org/Oryza_sativa/Info/Index).

Differentially expressed genes (DEGs) were analysed using DESeq2 (Love et al. 2014). Functional annotation of DEGs was performed using Swiss‐Prot, and the Kyoto Encyclopedia of Genes and Genomes (KEGG) database through the Majorbio online platform (www.majorbio.com). Genes showing a threshold of | log_2_ (fold change) | > 1 and a false discovery rate (FDR)‐adjusted *q* value < 0.05, calculated using the Benjamini‐Hochberg procedure, were considered significantly differentially expressed. Quantitative real‐time PCR (qRT‐PCR) was conducted using the SYBR Green system (TaKaRa, Dalian, China) for validation of the RNA‐Seq data. The 18S ribosomal RNA (18S) gene (Yan et al. 2023) was used as the internal reference gene amplified with the primer pair (18S‐F: CTACGTCCCTGCCCTTTCTACA and 18S‐R: ACACTTCACCGGACCATTCAA). Primers were designed using Primer Premier 5 software (http://www.PremierBiosoft.com/primerdesign/primerdesign.html). The relative expression levels of these genes were calculated using the 2^−ΔΔCt^ method.

Non‐targeted metabolomics was used to analyse a broad spectrum of metabolites the rice root (the same samples used for RNA‐seq analysis) and rhizosheath soil samples, using LC–MS/MS (Thermo Fisher, Waltham, Massachusetts, USA) by Majorbio (Majorbio Biotech Co. Ltd., Shanghai, China). Samples were freeze‐dried, ground to powder, and then 50 mg was extracted in 400 μL methanol (4:1, v/v) solution containing 0.02 mg mL^−1^ L‐2‐chlorophenylalanine as an internal standard. The mixture was settled at −10°C and processed with a High Throughput Tissue Crusher Wonbio‐96c (Shanghai Wanbo Biotechnology Co. Ltd., Shanghai, China) at 50 Hz for 6 min, followed by ultrasonication at 40 kHz for 30 min at 5°C. Samples were then stored at −20°C for 30 min to precipitate proteins. After centrifugation at 13 000 ***g*** for 15 min at 4°C, the supernatant was filtered through a 0.22‐μm microporous membrane and transferred to sample vials for LC–MS/MS analysis. Chromatographic separation of metabolites was performed on a Thermo UHPLC‐Q Exactive HF‐X system equipped with an ACQUITY BEH C_18_ column (100 mm × 2.1 mm, 1.7 μm; Waters, Milford, Massachusetts, USA). Mass spectrometry data were collected using a Thermo UHPLC‐Q Exactive Mass Spectrometer (Thermo Fisher, Waltham, Massachusetts, USA) with an electrospray ionisation (ESI) source operating in both positive and negative ion modes. The raw data were processed using Progenesis QI 2.3 (Nonlinear Dynamics, Waters, Milford, Massachusetts, USA) for peak detection and alignment. The R package “ropls” (Thévenot et al. 2015) was used to perform principal component analysis (PCA) and orthogonal least partial squares discriminant analysis (OPLS‐DA), and 7‐cycle interactive validation evaluating the stability of the model. Based on the OPLS‐DA model, metabolites with VIP (variable importance in projection) ≥ 1, and a false discovery rate (FDR)‐adjusted *q* value < 0.05 (Benjamini‐Hochberg correction) were identified as differentially expressed metabolites (DEMs). Metabolite classification and identification were based on the Human Metabolome Database (HMDB) (Wishart et al. 2018), and pathway enrichment was performed using the KEGG database. Weighted Gene Co‐expression Network Analysis (WGCNA) was conducted to identify modules of co‐expressed metabolites related to environmental factors (Langfelder and Horvath 2008). Heatmaps were generated to visualise metabolite enrichment across PI, PIR, and PIS treatments using the R package “pheatmap”. To investigate functional capacity of the rhizosheath microbial community, rhizosheath soil samples of the allelopathic rice grown alone or with barnyardgrass were subjected to shotgun metagenomics sequencing using an Illumina NovaSeq6000 sequencer (Illumina Inc., San Diego, CA, USA) at Majorbio Bio‐Pharm Technology Co. Ltd. (Shanghai, China). DNA was fragmented to an average size of about 400 bp using Covaris M220 (Gene Company Limited, Hong Kong, China) for paired‐end library construction. The paired‐end library was constructed using NEXTFLEX Rapid DNA‐Seq (Bioo Scientific, Austin, TX, USA), with adapters that contained the full complement of sequencing primer hybridization sites ligated to the blunt ends of fragments. Paired‐end Illumina reads were trimmed of adaptors and low‐quality reads (length < 50 bp, quality value < 20, or containing N bases) were removed using fastp (Chen et al. 2018). Metagenomics data were assembled using MEGAHIT78 (Li et al. 2015). Contigs of length ≥ 300 bp were selected as the final assembly results, and these contigs were used for further gene predictions and annotations. Open reading frames (ORFs) were predicted using Prodigal (Hyatt et al. 2010), retaining ORFs ≥ 100 bp. A non‐redundant gene catalogue was constructed with CD‐HIT (Fu et al. 2012) using 90% identity and coverage thresholds. Gene abundance was estimated using SOAPaligner (Li et al. 2008) with 95% identity. Taxonomic classification was performed by aligning genes to the NCBI NR database using DIAMOND (Buchfink et al. 2015), with *e*‐value cutoff 1*e*‐5. For functional annotation (KEGG Orthology, KOs) and taxonomic profiling (taxa), false discovery rate (FDR) correction using the Benjamini‐Hochberg procedure was applied wherever multiple hypothesis testing was performed. Community composition, KEGG pathway enrichment, and co‐occurrence network analyses were conducted via Majorbio and Gephi.

#### 4.3.2 Metabolomics Data Processing and Quality Control

Quality control (QC) samples were prepared by pooling equal aliquots of all samples and were injected at the beginning and end of the analytical sequence, as well as regularly throughout the run (one QC sample inserted after every nine analytical samples), to monitor instrument stability and data reproducibility. Raw LC–MS/MS data were preprocessed using the Majorbio metabolomics analysis pipeline. Features with missing values in more than 20% of samples within any group were removed. Remaining missing values were imputed using the minimum value method. Data were normalised using sum normalisation and log10‐transformed prior to downstream statistical analyses. Instrumental variation was assessed using QC samples, and only features with acceptable analytical reproducibility (QC relative standard deviation, RSD ≤ 30%) were retained for subsequent analyses. Signal drift correction was performed using the proprietary algorithm implemented in the Majorbio platform. Metabolite identification was conducted by matching accurate mass, retention time, and MS/MS spectra against public databases (HMDB and KEGG). Metabolite annotation confidence was reported according to the Metabolomics Standards Initiative (MSI), primarily at levels B(i) and B(ii).

#### 4.3.3 Integrated Transcriptome and Metabolome Analysis

To elucidate the coordinated responses of allelopathic rice to barnyardgrass stress, transcriptomic and metabolomic datasets were integrated. KEGG pathway enrichment analyses of DEGs and DEMs were performed to identify overlapping biological pathways affected by treatments. Correlation analyses between gene expression levels and metabolite abundances were conducted using Python SciPy and R.

### 4.4 Growth Inhibition Potential of Candidate Metabolites From Allelopathic Rice Root Tissue

The experiment was performed to evaluate the effect of the four selected amino acids, (2E)‐decenoyl‐ACP (traditional name: cycloleucine), L‐histidine, pipecolic acid, and 1‐aminocyclopropanecarboxylic acid from the allelopathic rice roots on barnyardgrass growth, and on the growth of two rice cultivars, allelopathic rice cv PI312777 (PI) and non‐allelopathic rice cv Tianyouhuazhan (TY). Analytical grade of the four amino acids was purchased from Macklin and aqueous solution prepared. Three mL of the solution at 0, 40, 80, 120 and 160 μM were evenly applied onto filter paper. These concentrations were selected based on a previous report (Bertin et al. 2007), who identified the nonprotein amino acid m‐tyrosine as a major phytotoxic compound released by fine fescue roots, with significant inhibitory effects on the growth of neighbouring plants. Barnyardgrass and rice seeds were germinated directly on the filter paper. Each petri dish contained 20 seedlings, and shoot and root lengths were measured 7 days after germination. For each concentration, three petri dishes were used as independent biological replicates (dish = replicate, *n* = 3), while individual seedlings within each dish were treated as subsamples. Control plants were grown on petri dishes treated with 3 mL of distilled water only. The entire experiment was repeated once with similar results. Shoot and root length of barnyardgrass and rice (20 seedlings per petri dish) were measured 7 days after germination and compared with the control plants grown on petri dishes treated only with 3 mL water. Each assay was performed in triplicates (total 50–60 seedlings) and repeated.

### 4.5 Statistical Analysis

PCA for amino acids and bacterial genera was performed to identify major axes of variation. Missing values in microbial variables were imputed using a PCA‐based approach implemented in the “missMDA” package, with the number of retained components estimated automatically using a regularised criterion; the robustness of downstream variation partitioning results was evaluated using stratified bootstrap resampling (*n* = 10 000). Variation partitioning analysis (VPA) was conducted to quantify the independent and shared contributions of amino acids and bacterial genera to plant‐available soil nitrogen using the “vegan” R package (Oksanen et al. 2020). Random forest analysis (Liaw and Wiener 2001) and permutation‐based significance testing (rfPermute) were used to identify key amino acids and bacterial genera influencing plant‐available soil nitrogen. Differentially expressed genes (DEGs) identified from the statistical analyses were subjected to KEGG pathway enrichment analysis. Enrichment was performed using Fisher's exact test, and *p*‐values were adjusted for multiple comparisons using the Benjamini–Hochberg false discovery rate (FDR) correction. Microbial abundance analysis and KEGG pathway enrichment between two groups were assessed by Student's *t*‐test. Multiple testing was corrected by FDR (Benjamini–Hochberg), and 95% confidence intervals were calculated using the inverted Student's *t* distribution. Amino acid bioassay data were analysed using linear mixed‐effects models with petri dish as a random effect, followed by Tukey's HSD tests. A one‐way analysis of variance (ANOVA) was used for all other differential analyses not otherwise specified, including comparisons of plant physiological traits, soil properties, gene expression, and KO abundances across treatments, followed by Tukey's honestly significant difference (HSD) tests at a significance level of *p* < 0.05. All data analyses and graphs were performed in R (version 4.0.3). Unless otherwise stated, data are presented as means ± standard deviation (SD); for mixed‐effects model analyses, results are shown as estimated marginal means ±95% confidence intervals.

## Author Contributions

S.L. designed the experiments, analysed the data, prepared most figures and tables, and wrote the manuscript. Q.Y. conducted the exogenous amino acids application experiment and qPCR. J.T. conducted the plant phytohormones determination. Y.L. wrote some methods and revised the manuscript. Q.P. designed the experiments, wrote the manuscript, and revised the manuscript. L.B. designed the experiments, provided critical suggestions, and revised the manuscript. All authors edited and approved the manuscript.

## Funding

This work was supported by National Natural Science Foundation of China, U22A20461, 32372564.

## Conflicts of Interest

The authors declare no conflicts of interest.

## Supporting information


**Table S1:** Summary of trimming and read mapping results of the sequences generated from allelopathic rice root under PI, PIR and PIS treatments.
**Table S2:** RT‐qPCR validation of the selected differentially expressed candidate contigs from the transcriptome dataset.
**Table S3:** Rhizosheath microorganisms significantly increased in rice under resistant and susceptible barnyardgrass stress.
**Table S4:** Representative KEGG orthologs (KOs) for quorum sensing and ABC transporter pathways and their leading bacterial taxa.
**Table S5:** Bootstrap‐based (*n* = 10 000) variation partitioning analysis (VPA) results assessing the robustness of bacterial and amino acid contributions.
**Table S6:** Amino acids used in the bioassays and their chemical information.
**Table S7:** Differentially expressed auxin‐ and ethylene‐related genes in allelopathic rice under resistant (R) versus susceptible (S) barnyardgrass stress.
**Figure S1:** Phenotypic comparison of flowering time between susceptible and resistant barnyardgrass grown under identical conditions. The resistant biotype shows earlier heading and flowering.
**Figure S2:** Weighted gene co‐expression network analysis (WGCNA) of all DAMs (Differentially Expressed Metabolites) with FPKM (Fragments Per Kilobase of transcript per Million mapped reads) > 1.
**Figure S3:** Heatmap of gene expression of the yellow module in roots, and the blue module in the rhizosheath soil. Pie chart displays counts of HMDB (Human Metabolome Database) taxonomy (subclass) for metabolites enriched in these modules.
**Figure S4:** Taxonomic classification of allelopathic rice rhizosheath soil microorganisms across different domains and their proportional representation.
**Figure S5:** PCoA (Principal Coordinates Analysis) analysis based on Bray–Curtis distances at the genus level, demonstrating differentiation of allelopathic rice rhizosheath microorganisms in response to barnyardgrass stress.
**Figure S6:** Correlation network diagram of bacteria and metabolites in barnyardgrass‐stress rice at the genus level.
**Figure S7:** Spearman rank correlations between bacterial abundance and plant‐available soil nitrogen, electrical conductivity (red and blue blocks indicate positive and negative correlation respectively).
**Figure S8:** Random forest analysis demonstrating the significance of amino acids and bacteria in regulating plant‐available soil nitrogen.
**Figure S9:** Principal component analysis (PCA) scree plots for amino acids and bacterial genera, showing the variance explained by each component.
**Figure S10:** Abundance of momilactone A, and arabinosyl‐glucosyl propanenitrile in the roots, 3,4‐dihydroxybutanenitrile glucoside in the rhizosheath soil of the allelopathic rice co‐cultured with barnyardgrass.
**Figure S11:** The *p* value for histogram of KEGG (Kyoto Encyclopedia of Genes and Genomes) enrichment analysis for integrated metabolomics and transcriptomics.
**Figure S12:** Dose response to quinclorac of barnyardgrass lines at 3 weeks after treatment.

## Data Availability

Data are available from the corresponding author upon request. Public availability is restricted due to ongoing patent applications and further research. All data necessary to support the conclusions are included in the article.

## References

[pbi70611-bib-0001] Aoki, D. , and H. Yamaguchi . 2008. “Genetic Relationship Between *Echinochloa crus‐galli* and *Echinochloa oryzicola* Accessions Inferred From Internal Transcribed Spacer and Chloroplast DNA Sequences.” Weed Biology and Management 8: 233–242. 10.1111/j.1445-6664.2008.00303.x.

[pbi70611-bib-0002] Bacilio‐Jiménez, M. , S. Aguilar‐Flores , E. Ventura‐Zapata , E. Pérez‐Campos , S. Bouquelet , and E. Zenteno . 2003. “Chemical Characterization of Root Exudates From Rice (*Oryza sativa*) and Their Effects on the Chemotactic Response of Endophytic Bacteria.” Plant and Soil 249: 271–277. 10.1023/A:1022888900465.

[pbi70611-bib-0003] Badri, D. V. , and J. M. Vivanco . 2009. “Regulation and Function of Root Exudates.” Plant, Cell & Environment 32: 666–681. 10.1111/j.1365-3040.2009.01926.x. 19143988

[pbi70611-bib-0004] Bajwa, A. A. , K. Jabran , M. Shahid , H. H. Ali , B. S. Chauhan , and Ehsanullah . 2015. “Eco‐Biology and Management of *Echinochloa crus‐galli* .” Crop Protection 75: 151–162. 10.1016/j.cropro.2015.06.001..

[pbi70611-bib-0005] Bauer, W. D. , and U. Mathesius . 2004. “Plant Responses to Bacterial Quorum Sensing Signals.” Current Opinion in Plant Biology 7: 429–433. 10.1016/j.pbi.2004.05.008. 15231266

[pbi70611-bib-0006] Berendsen, R. L. , G. Vismans , K. Yu , et al. 2018. “Disease‐Induced Assemblage of a Plant‐Beneficial Bacterial Consortium.” ISME Journal 12: 1496–1507. 10.1038/s41396-018-0093-1. 29520025 PMC5956071

[pbi70611-bib-0007] Bertin, C. , L. A. Weston , T. Huang , et al. 2007. “Grass Roots Chemistry: Meta‐Tyrosine, an Herbicidal Nonprotein Amino Acid.” Proceedings of the National Academy of Sciences 104: 16964–16969. 10.1073/pnas.0707198104. PMC2040483 17940026

[pbi70611-bib-0008] Bremner, J. M. 1965. “Inorganic Forms of Nitrogen.” In Methods of Soil Analysis: Part 2 Chemical and Microbiological Properties, 1179–1237. ASA‐SSSA. 10.2134/agronmonogr9.2.c33.

[pbi70611-bib-0009] Buchfink, B. , C. Xie , and D. H. Huson . 2015. “Fast and Sensitive Protein Alignment Using DIAMOND.” Nature Methods 12: 59–60. 10.1038/nmeth.3176. 25402007

[pbi70611-bib-0010] Carrión, V. J. , J. Perez‐Jaramillo , V. Cordovez , et al. 2019. “Pathogen‐Induced Activation of Disease‐Suppressive Functions in the Endophytic Root Microbiome.” Science 366: 606–612. 10.1126/science.aaw9285. 31672892

[pbi70611-bib-0011] Chen, S. , Y. Zhou , Y. Chen , and J. Gu . 2018. “Fastp: An Ultra‐Fast All‐In‐One FASTQ Preprocessor.” Bioinformatics 34: i884–i890. 10.1093/bioinformatics/bty560. 30423086 PMC6129281

[pbi70611-bib-0012] Cheng, F. , and Z. Cheng . 2015. “Research Progress on the Use of Plant Allelopathy in Agriculture and the Physiological and Ecological Mechanisms of Allelopathy.” Frontiers in Plant Science 6: 1020. 10.3389/fpls.2015.01020. 26635845 PMC4647110

[pbi70611-bib-0013] Chou, M.‐Y. , T. B. Andersen , M. E. Mechan Llontop , et al. 2023. “Terpenes Modulate Bacterial and Fungal Growth and Sorghum Rhizobiome Communities.” Microbiology Spectrum 11: e01332‐23. 10.1128/spectrum.01332-23. 37772854 PMC10580827

[pbi70611-bib-0014] Chroston, E. C. M. , N. Bziuk , E. J. Stauber , et al. 2024. “Plant Glucosinolate Biosynthesis and Breakdown Pathways Shape the Rhizosphere Bacterial/Archaeal Community.” Plant, Cell & Environment 47: 2127–2145. 10.1111/pce.14870. 38419355

[pbi70611-bib-0015] Coale, T. H. , V. Loconte , K. A. Turk‐Kubo , et al. 2024. “Nitrogen‐Fixing Organelle in a Marine Alga.” Science 384: 217–222. 10.1126/science.adk1075. 38603509

[pbi70611-bib-0016] Coleman, J. , M. Blake‐Kalff , and E. Davies . 1997. “Detoxification of Xenobiotics by Plants: Chemical Modification and Vacuolar Compartmentation.” Trends in Plant Science 2: 144–151. 10.1016/S1360-1385(97)01019-1.

[pbi70611-bib-0017] Conrath, U. , G. J. M. Beckers , C. J. G. Langenbach , and M. R. Jaskiewicz . 2015. “Priming for Enhanced Defense.” Annual Review of Phytopathology 53: 97–119. 10.1146/annurev-phyto-080614-120132. 26070330

[pbi70611-bib-0018] Cotton, T. E. A. , P. Pétriacq , D. D. Cameron , et al. 2019. “Metabolic Regulation of the Maize Rhizobiome by Benzoxazinoids.” ISME Journal 13: 1647–1658. 10.1038/s41396-019-0375-2. 30796337 PMC6592824

[pbi70611-bib-0019] Diao, F. , B. Jia , J. Luo , S. Ding , T. Liu , and W. Guo . 2024. “Arbuscular Mycorrhizal Fungi Drive Bacterial Community Assembly in Halophyte *Suaeda salsa* .” Microbiological Research 282: 127657. 10.1016/j.micres.2024.127657. 38422862

[pbi70611-bib-0110] Dilday, R. H. , J. D. Mattice , K. A. Moldenhauer , and W. Yan . 2001. “Allelopathic Potential in Rice Germplasm Against Ducksalad, Redstem and Barnyard Grass.” Journal of Crop Production 4, no. 2: 287–301. 10.1300/J144v04n02_11.

[pbi70611-bib-0020] Dinkeloo, K. , S. Boyd , and G. Pilot . 2018. “Update on Amino Acid Transporter Functions and on Possible Amino Acid Sensing Mechanisms in Plants.” Seminars in Cell & Developmental Biology 74: 105–113. 10.1016/j.semcdb.2017.05.021. 28705659

[pbi70611-bib-0021] Dudenhöffer, J.‐H. , S. Scheu , and A. Jousset . 2016. “Systemic Enrichment of Antifungal Traits in the Rhizosphere Microbiome After Pathogen Attack.” Journal of Ecology 104: 1566–1575. 10.1111/1365-2745.12626.

[pbi70611-bib-0022] Fu, L. , B. Niu , Z. Zhu , S. Wu , and W. Li . 2012. “CD‐HIT: Accelerated for Clustering the Next‐Generation Sequencing Data.” Bioinformatics 28: 3150–3152. 10.1093/bioinformatics/bts565. 23060610 PMC3516142

[pbi70611-bib-0023] Grossmann, K. 2010. “Auxin Herbicides: Current Status of Mechanism and Mode of Action.” Pest Management Science 66: 113–120. 10.1002/ps.1860. 19823992

[pbi70611-bib-0024] Grossmann, K. , and J. Kwiatkowski . 1993. “Selective Induction of Ethylene and Cyanide Biosynthesis Appears to Be Involved in the Selectivity of the Herbicide Quinclorac Between Rice and Barnyardgrass.” Journal of Plant Physiology 142: 457–466. 10.1016/S0176-1617(11)81252-6.

[pbi70611-bib-0025] Grossmann, K. , and J. Kwiatkowski . 2000. “The Mechanism of Quinclorac Selectivity in Grasses.” Pesticide Biochemistry and Physiology 66: 83–91. 10.1006/pest.1999.2461.

[pbi70611-bib-0026] Gulati, S. , M.‐B. Ballhausen , P. Kulkarni , R. Grosch , and P. Garbeva . 2020. “A Non‐Invasive Soil‐Based Setup to Study Tomato Root Volatiles Released by Healthy and Infected Roots.” Scientific Reports 10: 12704. 10.1038/s41598-020-69468-z. 32728091 PMC7391657

[pbi70611-bib-0027] Gunina, A. , and Y. Kuzyakov . 2015. “Sugars in Soil and Sweets for Microorganisms: Review of Origin, Content, Composition and Fate.” Soil Biology & Biochemistry 90: 87–100. 10.1016/j.soilbio.2015.07.021.

[pbi70611-bib-0028] Heap, I. 2024. “The International Herbicide‐Resistant Weed Database.” https://www.weedscience.org/Home.aspx.

[pbi70611-bib-0029] Heinemann, B. , and T. M. Hildebrandt . 2021. “The Role of Amino Acid Metabolism in Signaling and Metabolic Adaptation to Stress‐Induced Energy Deficiency in Plants.” Journal of Experimental Botany 72: 4634–4645. 10.1093/jxb/erab182. 33993299

[pbi70611-bib-0030] Helmke, P. A. , and D. L. Sparks . 1996. “Lithium, Sodium, Potassium, Rubidium, and Cesium.” In Methods of Soil Analysis: Part 3 Chemical Methods, 551–574. SSSA Book Series. 10.2136/sssabookser5.3.c19.

[pbi70611-bib-0031] Hennessy, J. E. , M. J. Latter , S. Fazelinejad , et al. 2018. “Hyperthermophilic Carbamate Kinase Stability and Anabolic In Vitro Activity at Alkaline pH.” Applied and Environmental Microbiology 84: e02250‐17. 10.1128/AEM.02250-17. 29150502 PMC5772250

[pbi70611-bib-0032] Hildebrandt, T. M. , A. Nunes Nesi , W. L. Araújo , and H.‐P. Braun . 2015. “Amino Acid Catabolism in Plants.” Molecular Plant 8: 1563–1579. 10.1016/j.molp.2015.09.005. 26384576

[pbi70611-bib-0033] Howden, A. J. M. , and G. M. Preston . 2009. “Nitrilase Enzymes and Their Role in Plant–Microbe Interactions.” Microbial Biotechnology 2: 441–451. 10.1111/j.1751-7915.2009.00111.x. 21255276 PMC3815905

[pbi70611-bib-0034] Hu, J. , C. Ricono , P. Fournier , S. Mondy , P. Vandenkoornhuyse , and C. Mony . 2023. “Neighbourhood Effect of Weeds on Wheat Root Endospheric Mycobiota.” Journal of Ecology 111: 994–1008. 10.1111/1365-2745.14073.

[pbi70611-bib-0035] Huang, A. C. , T. Jiang , Y.‐X. Liu , et al. 2019. “A Specialized Metabolic Network Selectively Modulates *Arabidopsis* Root Microbiota.” Science 364: eaau6389. 10.1126/science.aau6389. 31073042

[pbi70611-bib-0036] Hyatt, D. , G.‐L. Chen , P. F. LoCascio , M. L. Land , F. W. Larimer , and L. J. Hauser . 2010. “Prodigal: Prokaryotic Gene Recognition and Translation Initiation Site Identification.” BMC Bioinformatics 11: 119. 10.1186/1471-2105-11-119. 20211023 PMC2848648

[pbi70611-bib-0037] Isaac, R. A. , and W. C. Johnson . 1976. “Determination of Total Nitrogen in Plant Tissue, Using a Block Digestor.” Journal of the Association of Official Analytical Chemists 59: 98–100. 10.1093/jaoac/59.1.98. 6746471

[pbi70611-bib-0038] Jousset, A. , L. Rochat , A. Lanoue , M. Bonkowski , C. Keel , and S. Scheu . 2011. “Plants Respond to Pathogen Infection by Enhancing the Antifungal Gene Expression of Root‐Associated Bacteria.” Molecular Plant‐Microbe Interactions 24: 352–358. 10.1094/MPMI-09-10-0208. 21077773

[pbi70611-bib-0039] Kato‐Noguchi, H. 2011. “Barnyard Grass‐Induced Rice Allelopathy and Momilactone B.” Journal of Plant Physiology 168: 1016–1020. 10.1016/j.jplph.2010.12.021. 21392842

[pbi70611-bib-0040] Kato‐Noguchi, H. , M. Hasegawa , T. Ino , K. Ota , and H. Kujime . 2010. “Contribution of Momilactone A and B to Rice Allelopathy.” Journal of Plant Physiology 167: 787–791. 10.1016/j.jplph.2010.01.014. 20170980

[pbi70611-bib-0041] Kato‐Noguchi, H. , and T. Ino . 2003. “Rice Seedlings Release Momilactone B Into the Environment.” Phytochemistry 63: 551–554. 10.1016/S0031-9422(03)00194-8. 12809715

[pbi70611-bib-0042] Kato‐Noguchi, H. , and T. Ino . 2013. “The Chemical‐Mediated Allelopathic Interaction Between Rice and Barnyard Grass.” Plant and Soil 370: 267–275. 10.1007/s11104-012-1322-4.

[pbi70611-bib-0043] Kerchev, P. I. , B. Fenton , C. H. Foyer , and R. D. Hancock . 2012. “Plant Responses to Insect Herbivory: Interactions Between Photosynthesis, Reactive Oxygen Species and Hormonal Signalling Pathways.” Plant, Cell & Environment 35: 441–453. 10.1111/j.1365-3040.2011.02399.x. 21752032

[pbi70611-bib-0044] Kim, D. , B. Langmead , and S. L. Salzberg . 2015. “HISAT: A Fast Spliced Aligner With Low Memory Requirements.” Nature Methods 12: 357–360. 10.1038/nmeth.3317. 25751142 PMC4655817

[pbi70611-bib-0045] Kiyama, H. , A. Matsunaga , G. Suzuki , and K. Gomi . 2021. “Monoterpene Geraniol Produced by Rice Terpene Synthase 21 Suppresses the Expression of Cell‐Division Related Genes in the Rice Bacterial Pathogen, *Xanthomonas oryzae* Pv. *oryzae* .” Physiological and Molecular Plant Pathology 115: 101673. 10.1016/j.pmpp.2021.101673.

[pbi70611-bib-0046] Kong, C. , X. Xu , B. Zhou , F. Hu , C. Zhang , and M. Zhang . 2004. “Two Compounds From Allelopathic Rice Accession and Their Inhibitory Activity on Weeds and Fungal Pathogens.” Phytochemistry 65: 1123–1128. 10.1016/j.phytochem.2004.02.017.. 15110693

[pbi70611-bib-0047] Kong, C.‐H. , T. D. Xuan , T. D. Khanh , H.‐D. Tran , and N. T. Trung . 2019. “Allelochemicals and Signaling Chemicals in Plants.” Molecules 24: 2737. 10.3390/molecules24152737. 31357670 PMC6695906

[pbi70611-bib-0048] Kong, C.‐H. , S.‐Z. Zhang , Y.‐H. Li , et al. 2018. “Plant Neighbor Detection and Allelochemical Response Are Driven by Root‐Secreted Signaling Chemicals.” Nature Communications 9: 3867. 10.1038/s41467-018-06429-1. PMC6155373 30250243

[pbi70611-bib-0049] Kostylev, M. , D. Y. Kim , N. E. Smalley , I. Salukhe , E. P. Greenberg , and A. A. Dandekar . 2019. “Evolution of the *Pseudomonas aeruginosa* Quorum‐Sensing Hierarchy.” Proceedings of the National Academy of Sciences 116: 7027–7032. 10.1073/pnas.1819796116. PMC6452656 30850547

[pbi70611-bib-0050] Langfelder, P. , and S. Horvath . 2008. “WGCNA: An R Package for Weighted Correlation Network Analysis.” BMC Bioinformatics 9: 559. 10.1186/1471-2105-9-559. 19114008 PMC2631488

[pbi70611-bib-0051] Lendzemo, V. W. , T. W. Kuyper , R. Matusova , H. J. Bouwmeester , and A. V. Ast . 2007. “Colonization by Arbuscular Mycorrhizal Fungi of Sorghum Leads to Reduced Germination and Subsequent Attachment and Emergence of *Striga hermonthica* .” Plant Signaling & Behavior 2: 58–62. 10.4161/psb.2.1.3884. 19516969 PMC2633899

[pbi70611-bib-0052] Leschine, S. B. 1995. “Cellulose Degradation in Anaerobic Environments.” Annual Review of Microbiology 49: 399–426. 10.1146/annurev.mi.49.100195.002151. 8561466

[pbi70611-bib-0053] Li, B. , and C. N. Dewey . 2011. “RSEM: Accurate Transcript Quantification From RNA‐Seq Data With or Without a Reference Genome.” BMC Bioinformatics 12: 323. 10.1186/1471-2105-12-323. 21816040 PMC3163565

[pbi70611-bib-0054] Li, D. , C.‐M. Liu , R. Luo , K. Sadakane , and T.‐W. Lam . 2015. “MEGAHIT: An Ultra‐Fast Single‐Node Solution for Large and Complex Metagenomics Assembly via Succinct de Bruijn Graph.” Bioinformatics 31: 1674–1676. 10.1093/bioinformatics/btv033. 25609793

[pbi70611-bib-0055] Li, L.‐L. , H.‐H. Zhao , and C.‐H. Kong . 2020. “(−)‐Loliolide, the Most Ubiquitous Lactone, Is Involved in Barnyardgrass‐Induced Rice Allelopathy.” Journal of Experimental Botany 71: 1540–1550. 10.1093/jxb/erz497. 31677347

[pbi70611-bib-0056] Li, R. , Y. Li , K. Kristiansen , and J. Wang . 2008. “SOAP: Short Oligonucleotide Alignment Program.” Bioinformatics 24: 713–714. 10.1093/bioinformatics/btn025. 18227114

[pbi70611-bib-0057] Li, S. , Y. Wang , L. Bai , and Q. Peng . 2020. “Effects of Different Densities of Resistant and Sensitive Barnyardgrass on Nitrogen Content and the Photosynthesis in Rice.” Journal of Plant Physiology 56: 2677–2682. 10.13592/j.cnki.ppj.2020.0305.

[pbi70611-bib-0058] Li, Y. , X. Jian , Y. Li , et al. 2020. “ *OsPAL2‐1* Mediates Allelopathic Interactions Between Rice and Specific Microorganisms in the Rhizosphere Ecosystem.” Frontiers in Microbiology 11: 1411. 10.3389/fmicb.2020.01411. 32793125 PMC7391800

[pbi70611-bib-0059] Li, Y.‐H. , Z.‐C. Xia , and C.‐H. Kong . 2016. “Allelobiosis in the Interference of Allelopathic Wheat With Weeds.” Pest Management Science 72: 2146–2153. 10.1002/ps.4246. 26833449

[pbi70611-bib-0060] Liaw, A. , and M. Wiener . 2001. “Classification and Regression by RandomForest.” Forest Research News 3: 18–22.

[pbi70611-bib-0061] Liu, H. , J. Li , L. C. Carvalhais , et al. 2021. “Evidence for the Plant Recruitment of Beneficial Microbes to Suppress Soil‐Borne Pathogens.” New Phytologist 229: 2873–2885. 10.1111/nph.17057. 33131088

[pbi70611-bib-0062] Lorenzo, P. , C. S. Pereira , and S. Rodríguez‐Echeverría . 2013. “Differential Impact on Soil Microbes of Allelopathic Compounds Released by the Invasive *Acacia dealbata* Link.” Soil Biology & Biochemistry 57: 156–163. 10.1016/j.soilbio.2012.08.018.

[pbi70611-bib-0063] Love, M. I. , W. Huber , and S. Anders . 2014. “Moderated Estimation of Fold Change and Dispersion for RNA‐Seq Data With DESeq2.” Genome Biology 15: 550. 10.1186/s13059-014-0550-8. 25516281 PMC4302049

[pbi70611-bib-0064] Macías, F. A. , F. J. Mejías , and J. M. Molinillo . 2019. “Recent Advances in Allelopathy for Weed Control: From Knowledge to Applications.” Pest Management Science 75: 2413–2436. 10.1002/ps.5355. 30684299

[pbi70611-bib-0065] Mauch‐Mani, B. , I. Baccelli , E. Luna , and V. Flors . 2017. “Defense Priming: An Adaptive Part of Induced Resistance.” Annual Review of Plant Biology 68: 485–512. 10.1146/annurev-arplant-042916-041132. 28226238

[pbi70611-bib-0066] Moormann, J. , B. Heinemann , and T. M. Hildebrandt . 2022. “News About Amino Acid Metabolism in Plant–Microbe Interactions.” Trends in Biochemical Sciences 47: 839–850. 10.1016/j.tibs.2022.07.001. 35927139

[pbi70611-bib-0067] Murphy, K. M. , J. Edwards , K. B. Louie , et al. 2021. “Bioactive Diterpenoids Impact the Composition of the Root‐Associated Microbiome in Maize (*Zea mays*).” Scientific Reports 11: 333. 10.1038/s41598-020-79320-z. 33431904 PMC7801432

[pbi70611-bib-0068] Nelson, D. W. , and L. E. Sommers . 1996. “Total Carbon, Organic Carbon, and Organic Matter.” In Methods of Soil Analysis, 961–1010. John Wiley & Sons, Ltd. 10.2136/sssabookser5.3.c34DigitalObjectIdentifier(DOI).

[pbi70611-bib-0069] Oksanen, J. , F. G. Blanchet , M. Friendly , R. Kindt , P. Legendre , and D. McGlinn . 2020. “vegan Community Ecology Package Version 2.5‐7.” https://CRAN.R‐project.org/package=vegan.

[pbi70611-bib-0070] Olsen, S. R. 1954. Estimation of Available Phosphorus in Soils by Extraction With Sodium Bicarbonate. USDA Circular No. 939. US Government Printing Office.

[pbi70611-bib-0071] Pang, J. , M. H. Ryan , K. H. M. Siddique , and R. J. Simpson . 2017. “Unwrapping the Rhizosheath.” Plant and Soil 418: 129–139. 10.1007/s11104-017-3358-y.

[pbi70611-bib-0072] Paschalidis, K. , G. Tsaniklidis , B.‐Q. Wang , et al. 2019. “The Interplay Among Polyamines and Nitrogen in Plant Stress Responses.” Plants 8: 315. 10.3390/plants8090315. 31480342 PMC6784213

[pbi70611-bib-0073] Peng, Q. , H. Han , X. Yang , L. Bai , Q. Yu , and B. P. Stephen . 2019. “Quinclorac Resistance in *Echinochloa crus‐galli* From China.” Rice Science 26: 300–308. 10.1016/j.rsci.2019.08.004.

[pbi70611-bib-0074] Pertea, M. , G. M. Pertea , C. M. Antonescu , T.‐C. Chang , J. T. Mendell , and S. L. Salzberg . 2015. “StringTie Enables Improved Reconstruction of a Transcriptome From RNA‐Seq Reads.” Nature Biotechnology 33: 290–295. 10.1038/nbt.3122. PMC4643835 25690850

[pbi70611-bib-0075] Pieterse, C. M. J. , C. Zamioudis , R. L. Berendsen , D. M. Weller , S. C. M. V. Wees , and P. A. H. M. Bakker . 2014. “Induced Systemic Resistance by Beneficial Microbes.” Annual Review of Phytopathology 52: 347–375. 10.1146/annurev-phyto-082712-102340. 24906124

[pbi70611-bib-0076] Pineda, A. , S.‐J. Zheng , J. J. A. van Loon , C. M. J. Pieterse , and M. Dicke . 2010. “Helping Plants to Deal With Insects: The Role of Beneficial Soil‐Borne Microbes.” Trends in Plant Science 15: 507–514. 10.1016/j.tplants.2010.05.007. 20542720

[pbi70611-bib-0077] Quiñones, B. , G. Dulla , and S. E. Lindow . 2005. “Quorum Sensing Regulates Exopolysaccharide Production, Motility, and Virulence in *Pseudomonas syringae* .” Molecular Plant‐Microbe Interactions 18: 682–693. 10.1094/MPMI-18-0682. 16042014

[pbi70611-bib-0078] Rafique, M. , A. Ali , M. Naveed , et al. 2022. “Deciphering the Potential Role of Symbiotic Plant Microbiome and Amino Acid Application on Growth Performance of Chickpea Under Field Conditions.” Frontiers in Plant Science 13: 852851. 10.3389/fpls.2022.852851. 35646024 PMC9134094

[pbi70611-bib-0079] Rhoades, J. d. 1982. “Cation Exchange Capacity.” In Methods of Soil Analysis: Part 2 Chemical and Microbiological Properties, 2nd ed., 149–157. American Society of Agronomy. 10.2134/agronmonogr9.2.2ed.c8.

[pbi70611-bib-0080] Schellenberger, S. , H. L. Drake , and S. Kolb . 2012. “Impairment of Cellulose‐ and Cellobiose‐Degrading Soil Bacteria by Two Acidic Herbicides.” FEMS Microbiology Letters 327: 60–65. 10.1111/j.1574-6968.2011.02460.x. 22098368

[pbi70611-bib-0081] Senthilkumar, M. , N. Amaresan , and A. Sankaranarayanan . 2021. “Estimation of Glutamine Synthetase (GS), glutamate Synthase (GOGAT), and Glucose Dehydrogenase (GDH).” In Plant‐Microbe Interactions: Laboratory Techniques, edited by M. Senthilkumar , N. Amaresan , and A. Sankaranarayanan , 45–47. Springer US. 10.1007/978-1-0716-1080-0_7.

[pbi70611-bib-0082] Shiade, S. R. G. , A. Zand‐Silakhoor , A. Fathi , et al. 2024. “Plant Metabolites and Signaling Pathways in Response to Biotic and Abiotic Stresses: Exploring Bio Stimulant Applications.” Plant Stress 12: 100454. 10.1016/j.stress.2024.100454.

[pbi70611-bib-0083] Shimura, K. , A. Okada , K. Okada , et al. 2007. “Identification of a Biosynthetic Gene Cluster in Rice for Momilactones.” Journal of Biological Chemistry 282: 34013–34018. 10.1074/jbc.M703344200. 17872948

[pbi70611-bib-0084] Shirsat, S. , and K. Suthindhiran . 2025. “Preparation and Evaluation of *Magnetospirillum gryphiswaldense* MSR‐1 Bioinoculant on the Growth and Productivity of *Vigna radiata* (L.) R. Wilczek.” 3 Biotech 15: 49. 10.1007/s13205-025-04206-8. PMC11751356 39850094

[pbi70611-bib-0085] Simmons, T. , D. F. Caddell , S. Deng , and D. Coleman‐Derr . 2018. “Exploring the Root Microbiome: Extracting Bacterial Community Data From the Soil, Rhizosphere, and Root Endosphere.” Journal of Visualized Experiments 2: 57561. 10.3791/57561. PMC6101100 29782021

[pbi70611-bib-0086] Singh, S. , G. Bashri , A. Singh , and S. M. Prasad . 2016. “Regulation of Xenobiotics in Higher Plants: Signalling and Detoxification.” In Plant Responses to Xenobiotics, edited by A. Singh , S. M. Prasad , and R. P. Singh , 39–56. Springer. 10.1007/978-981-10-2860-1_3.

[pbi70611-bib-0087] Soares, A. R. , R. Marchiosi , R. d. C. Siqueira‐Soares , R. de Barbosa Lima , W. dos Dantas Santos , and O. Ferrarese‐Filho . 2014. “The Role of L‐DOPA in Plants.” Plant Signaling & Behavior 9: e28275. 10.4161/psb.28275. 24598311 PMC4091518

[pbi70611-bib-0088] Steindler, L. , I. Bertani , L. De Sordi , S. Schwager , L. Eberl , and V. Venturi . 2009. “LasI/R and RhlI/R Quorum Sensing in a Strain of *Pseudomonas aeruginosa* Beneficial to Plants.” Applied and Environmental Microbiology 75: 5131–5140. 10.1128/AEM.02914-08. 19525275 PMC2725484

[pbi70611-bib-0089] Sultana, M. H. , M. Alamin , J. Qiu , L. Fan , and C. Ye . 2023. “Transcriptomic Profiling Reveals Candidate Allelopathic Genes in Rice Responsible for Interactions With Barnyardgrass.” Frontiers in Plant Science 14: 1104951. 10.3389/fpls.2023.1104951. 36875579 PMC9982016

[pbi70611-bib-0090] Thévenot, E. A. , A. Roux , Y. Xu , E. Ezan , and C. Junot . 2015. “Analysis of the Human Adult Urinary Metabolome Variations With Age, Body Mass Index, and Gender by Implementing a Comprehensive Workflow for Univariate and OPLS Statistical Analyses.” Journal of Proteome Research 14: 3322–3335. 10.1021/acs.jproteome.5b00440. 26088811

[pbi70611-bib-0091] Thives Santos, W. , V. Dwivedi , H. Ngoc Duong , et al. 2024. “Mechanism of Action of the Toxic Proline Mimic Azetidine 2‐Carboxylic Acid in Plants.” Plant Journal 120: 2904–2918. 10.1111/tpj.17154. 39625042

[pbi70611-bib-0092] Trivedi, P. , J. E. Leach , S. G. Tringe , T. Sa , and B. K. Singh . 2020. “Plant‐Microbiome Interactions: From Community Assembly to Plant Health.” Nature Reviews Microbiology 18: 607–621. 10.1038/s41579-020-0412-1. 32788714

[pbi70611-bib-0093] Vivanco, J. M. , H. P. Bais , F. R. Stermitz , G. C. Thelen , and R. M. Callaway . 2004. “Biogeographical Variation in Community Response to Root Allelochemistry: Novel Weapons and Exotic Invasion.” Ecology Letters 7: 285–292. 10.1111/j.1461-0248.2004.00576.x.

[pbi70611-bib-0094] Voges, M. J. E. E. E. , Y. Bai , P. Schulze‐Lefert , and E. S. Sattely . 2019. “Plant‐Derived Coumarins Shape the Composition of an Arabidopsis Synthetic Root Microbiome.” Proceedings of the National Academy of Sciences of the United States of America 116: 12558–12565. 10.1073/pnas.1820691116. 31152139 PMC6589675

[pbi70611-bib-0095] Vurro, M. , A. Boari , A. L. Pilgeram , and D. C. Sands . 2006. “Exogenous Amino Acids Inhibit Seed Germination and Tubercle Formation by *Orobanche ramosa* (Broomrape): Potential Application for Management of Parasitic Weeds.” Biological Control 36: 258–265. 10.1016/j.biocontrol.2005.09.017.

[pbi70611-bib-0096] Wang, Q. , M. L. Hillwig , and R. J. Peters . 2011. “ *CYP99A3*: Functional Identification of a Diterpene Oxidase From the Momilactone Biosynthetic Gene Cluster in Rice.” Plant Journal 65: 87–95. 10.1111/j.1365-313X.2010.04408.x. PMC3735987 21175892

[pbi70611-bib-0097] Wishart, D. S. , Y. D. Feunang , A. Marcu , et al. 2018. “HMDB 4.0: The Human Metabolome Database for 2018.” Nucleic Acids Research 46: D608–D617. 10.1093/nar/gkx1089. 29140435 PMC5753273

[pbi70611-bib-0098] Xuan, T. D. , S. Tawata , T. D. Khanh , T. D. Xuan , S. Tawata , and T. D. Khanh . 2013. “Herbicidal Activity of Mimosine and Its Derivatives.” In Herbicides—Advances in Research, 299–312. IntechOpen. 10.5772/55845.

[pbi70611-bib-0099] Yan, Q. , J. Tong , S. Li , and Q. Peng . 2023. “Barnyard Grass Stress Triggers Changes in Root Traits and Phytohormone Levels in Allelopathic and Non‐Allelopathic Rice.” Biology 12: 1074. 10.3390/biology12081074. 37626960 PMC10452299

[pbi70611-bib-0100] Yang, X. , H. Han , J. Cao , Y. Li , Q. Yu , and S. B. Powles . 2021. “Exploring Quinclorac Resistance Mechanisms in From China.” Pest Management Science 77: 194–201. 10.1002/ps.6007. 32652760

[pbi70611-bib-0101] Yang, X.‐F. , C.‐H. Kong , X. Yang , and Y.‐F. Li . 2017. “Interference of Allelopathic Rice With Penoxsulam‐Resistant Barnyardgrass.” Pest Management Science 73: 2310–2317. 10.1002/ps.4617. 28523765

[pbi70611-bib-0102] Yang, X.‐F. , L.‐L. Li , Y. Xu , and C.‐H. Kong . 2018. “Kin Recognition in Rice ( *Oryza sativa* ) Lines.” New Phytologist 220: 567–578. 10.1111/nph.15296. 29956839

[pbi70611-bib-0103] You, L.‐X. , P. Wang , and C.‐H. Kong . 2011. “The Levels of Jasmonic Acid and Salicylic Acid in a Rice‐Barnyardgrass Coexistence System and Their Relation to Rice Allelochemicals.” Biochemical Systematics and Ecology 39: 491–497. 10.1016/j.bse.2011.07.007.

[pbi70611-bib-0104] Young, J. M. , S. B. Leschine , and G. Reguera . 2012. “Reversible Control of Biofilm Formation by *Cellulomonas* spp. in Response to Nitrogen Availability.” Environmental Microbiology 14: 594–604. 10.1111/j.1462-2920.2011.02596.x. 21951594

[pbi70611-bib-0105] Yu, P. , X. He , M. Baer , et al. 2021. “Plant Flavones Enrich Rhizosphere *Oxalobacteraceae* to Improve Maize Performance Under Nitrogen Deprivation.” Nature Plants 7: 481–499. 10.1038/s41477-021-00897-y. 33833418

[pbi70611-bib-0106] Zayed, O. , O. A. Hewedy , A. Abdelmoteleb , et al. 2023. “Nitrogen Journey in Plants: From Uptake to Metabolism, Stress Response, and Microbe Interaction.” Biomolecules 13: 1443. 10.3390/biom13101443. 37892125 PMC10605003

[pbi70611-bib-0108] Zhang, Z. , J. Cao , T. Gu , X. Yang , and Y. Li . 2021. “Co‐Planted Barnyardgrass Reduces Rice Yield by Inhibiting Plant Above‐and Belowground‐Growth During Post‐Heading Stages.” Crop Journal 9: 1198–1207. 10.1016/j.cj.2020.10.011.

[pbi70611-bib-0107] Zhang, Z. , T. Gu , B. Zhao , et al. 2017. “Effects of Common *Echinochloa* Varieties on Grain Yield and Grain Quality of Rice.” Field Crops Research 203: 163–172. 10.1016/j.fcr.2016.12.003.

[pbi70611-bib-0109] Zheng, Y.‐L. , Y.‐L. Feng , L.‐K. Zhang , et al. 2015. “Integrating Novel Chemical Weapons and Evolutionarily Increased Competitive Ability in Success of a Tropical Invader.” New Phytologist 205: 1350–1359. 10.1111/nph.13135. 25367824

