# Supplementary material for: Distinct Defence Mechanisms of Allelopathic Rice Against Quinclorac‐Susceptible and ‐Resistant Barnyardgrass: Involvement of Specific Metabolites and Rhizosheath Microbiota
Source: Plant Biotechnol J. 2026 Feb 25;24(6):3876–96. doi: 10.1111/pbi.70611 (PMC13205853; doi:10.1111/pbi.70611)
Supplement: Supplementary file 1 — Table S1: Summary of trimming and read mapping results of the sequences generated from allelopathic rice root under PI, PIR and PIS treatments. Table S2: RT‐qPCR validation of the selected differentially expressed candidate contigs from the transcriptome dataset. Table S3: Rhizosheath microorganisms significantly increased in rice under resistant and susceptible barnyardgrass stress. Table S4: Representative KEGG orthologs (KOs) for quorum sensing and ABC transporter pathways and their leading bacterial taxa. Table S5: Bootstrap‐based (n = 10 000) variation partitioning analysis (VPA) results assessing the robustness of bacterial and amino acid contributions. Table S6: Amino acids used in the bioassays and their chemical information. Table S7: Differentially expressed auxin‐ and ethylene‐related genes in allelopathic rice under resistant (R) versus susceptible (S) barnyardgrass stress. Figure S1: Phenotypic comparison of flowering time between susceptible and resistant barnyardgrass grown under identical conditions. The resistant biotype shows earlier heading and flowering. Figure S2: Weighted gene co‐expression network analysis (WGCNA) of all DAMs (Differentially Expressed Metabolites) with FPKM (Fragments Per Kilobase of transcript per Million mapped reads) > 1. Figure S3: Heatmap of gene expression of the yellow module in roots, and the blue module in the rhizosheath soil. Pie chart displays counts of HMDB (Human Metabolome Database) taxonomy (subclass) for metabolites enriched in these modules. Figure S4: Taxonomic classification of allelopathic rice rhizosheath soil microorganisms across different domains and their proportional representation. Figure S5: PCoA (Principal Coordinates Analysis) analysis based on Bray–Curtis distances at the genus level, demonstrating differentiation of allelopathic rice rhizosheath microorganisms in response to barnyardgrass stress. Figure S6: Correlation network diagram of bacteria and metabolites in barnyardgrass‐stress rice at [file PBI-24-3876-s001.zip › Supplementary_Figures_and_Tables_Cleaned.docx]

Table S1. Summary of trimming and read mapping results of the sequences generated from allelopathic rice root under PI, PIR and PIS treatments. PI, PIR and PIS represent monocultured allelopathic rice with no barnyardgrass, allelopathic rice co-cultured with quinclorac-resistant and -susceptible barnyardgrass, respectively.

| Sample | Raw reads | Raw bases | Clean reads | Clean bases | Error rate (%) | Q20(%) | Q30(%) | GC content (%) |
| --- | --- | --- | --- | --- | --- | --- | --- | --- |
| PI | 42332206 | 6392163106 | 41780636 | 6282161154 | 0.0254 | 97.77 | 93.82 | 50.36 |
| PI | 52093836 | 7866169236 | 51426800 | 7741926972 | 0.0255 | 97.77 | 93.74 | 50.8 |
| PI | 41424834 | 6255149934 | 40889176 | 6150787049 | 0.0251 | 97.9 | 94.17 | 51.31 |
| PI | 43566152 | 6578488952 | 42872018 | 6444389974 | 0.0253 | 97.81 | 93.99 | 51.81 |
| PI | 52240192 | 7888268992 | 51555260 | 7752626956 | 0.0252 | 97.86 | 94.06 | 51.06 |
| PI | 41849880 | 6319331880 | 41335094 | 6220651348 | 0.0251 | 97.89 | 94.12 | 51.04 |
| PIR | 48261390 | 7287469890 | 47595034 | 7153134483 | 0.0251 | 97.91 | 94.2 | 50.84 |
| PIR | 53381244 | 8060567844 | 52669014 | 7922083023 | 0.0252 | 97.86 | 94.01 | 50.67 |
| PIR | 44844550 | 6771527050 | 44283028 | 6658606776 | 0.0253 | 97.83 | 93.91 | 50.47 |
| PIR | 51995570 | 7851331070 | 51180730 | 7689937919 | 0.0252 | 97.88 | 94.1 | 50.29 |
| PIR | 44133338 | 6664134038 | 43398808 | 6518145860 | 0.025 | 97.94 | 94.3 | 50.67 |
| PIR | 42381194 | 6399560294 | 41715244 | 6273590153 | 0.025 | 97.96 | 94.29 | 50.38 |
| PIS | 41718146 | 6299440046 | 41222282 | 6197032197 | 0.0254 | 97.82 | 93.88 | 50.5 |
| PIS | 55777994 | 8422477094 | 54949298 | 8272759256 | 0.0257 | 97.67 | 93.52 | 50.81 |
| PIS | 44833800 | 6769903800 | 44247600 | 6651115874 | 0.025 | 97.94 | 94.26 | 51.1 |
| PIS | 45483886 | 6868066786 | 44817554 | 6733350306 | 0.0251 | 97.88 | 94.18 | 51.44 |
| PIS | 50136500 | 7570611500 | 49417602 | 7433459457 | 0.0253 | 97.82 | 93.97 | 51.46 |
| PIS | 47873486 | 7228896386 | 47249570 | 7107231177 | 0.0252 | 97.85 | 94 | 51.03 |

Table S2. RT-qPCR validation of the selected differentially expressed candidate contigs from the transcriptome dataset. PIR and PIS represent allelopathic rice co-cultured with quinclorac-resistant and -susceptible barnyardgrass, respectively. * represents *P* < 0.05.

| Contig | Annotation | Relative expression level  PIR PIS | |
| --- | --- | --- | --- |
| *Os07g0104500* | peroxidase function | 2.28 ^*^ | 1.83 ^*^ |
| *Os03g0368000* | peroxidase function | 1.70 ^*^ | 1.80 ^*^ |
| *Os05g0158600* | GA biosynthesis | 1.58 ^*^ | 1.40 |
| *Os05g0208550* | GA biosynthesis | 2.00 ^*^ | 0.17 |
| *Os04g0522500* | GA biosynthesis | 1.71 ^*^ | 0.75 |
| *Os04g0543900* | nitrogen metabolism | 3.02 ^*^ | 1.14 |
| *Os04g0178300* | momilactone A biosynthesis | 0.15 | 3.97 ^*^ |
| *Os04g0178400* | momilactone A biosynthesis | 0.21 | 3.68 ^*^ |
| *Os04g0179200* | momilactone A biosynthesis | 0.57 | 9.74 ^*^ |
| *Os11g0182200* | flavonoid biosynthesis | 3.17 ^*^ | 1.61 |
| *Os07g0592600* | plant hormone signal transduction | 1.86 ^*^ | 1.00 |
| *Os09g0451400* | amino acids metabolism | 1.90 ^*^ | 1.47 |

Table S5. Bootstrap-based (n = 10,000) variation partitioning analysis (VPA) results assessing the robustness of bacterial and amino acid contributions

|  | **Mean ± SD** | **95% CI** |
| --- | --- | --- |
| Bacteria only | 0.110 ± 0.059 | (0.007, 0.235) |
| Amino acids only | −0.005 ± 0.014 | (−0.021, 0.033) |
| Shared | 0.763 ± 0.072 | (0.609, 0.885) |
| Residual | 0.132 ± 0.052 | (0.041, 0.244) |


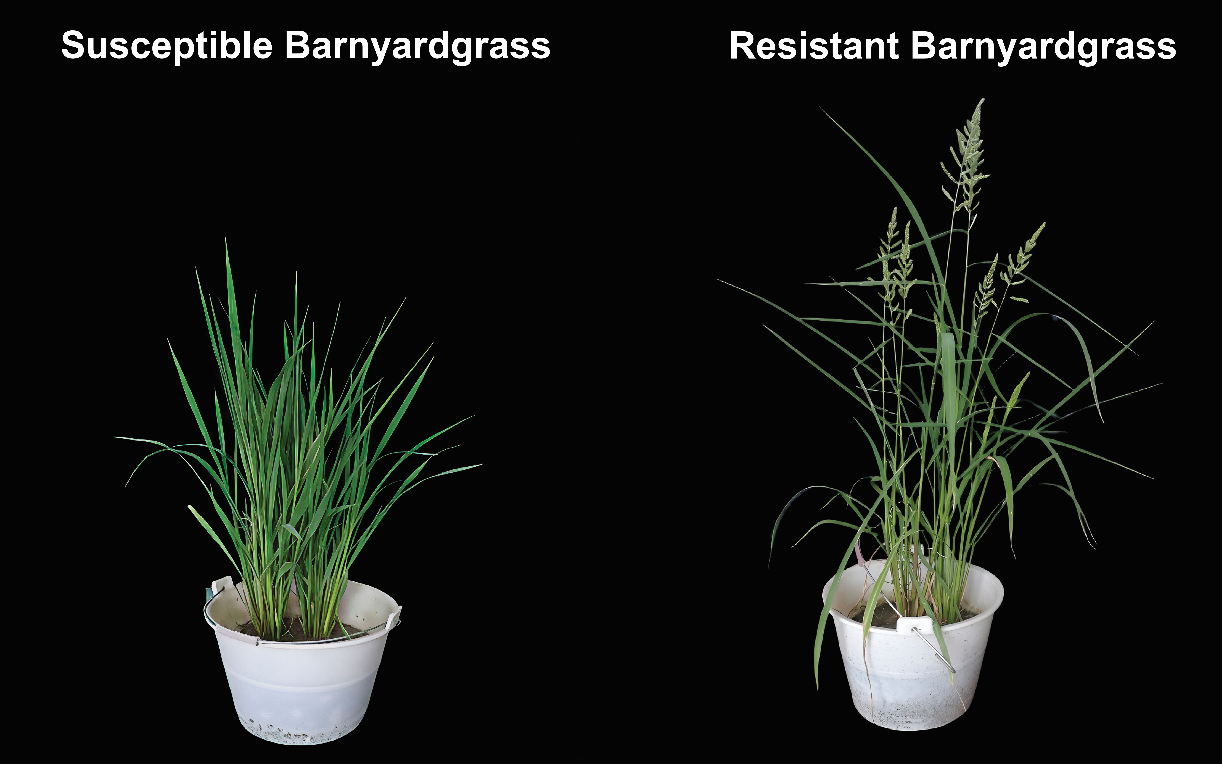


Figure S1. Phenotypic comparison of flowering time between susceptible and resistant barnyardgrass grown under identical conditions. The resistant biotype shows earlier heading and flowering.


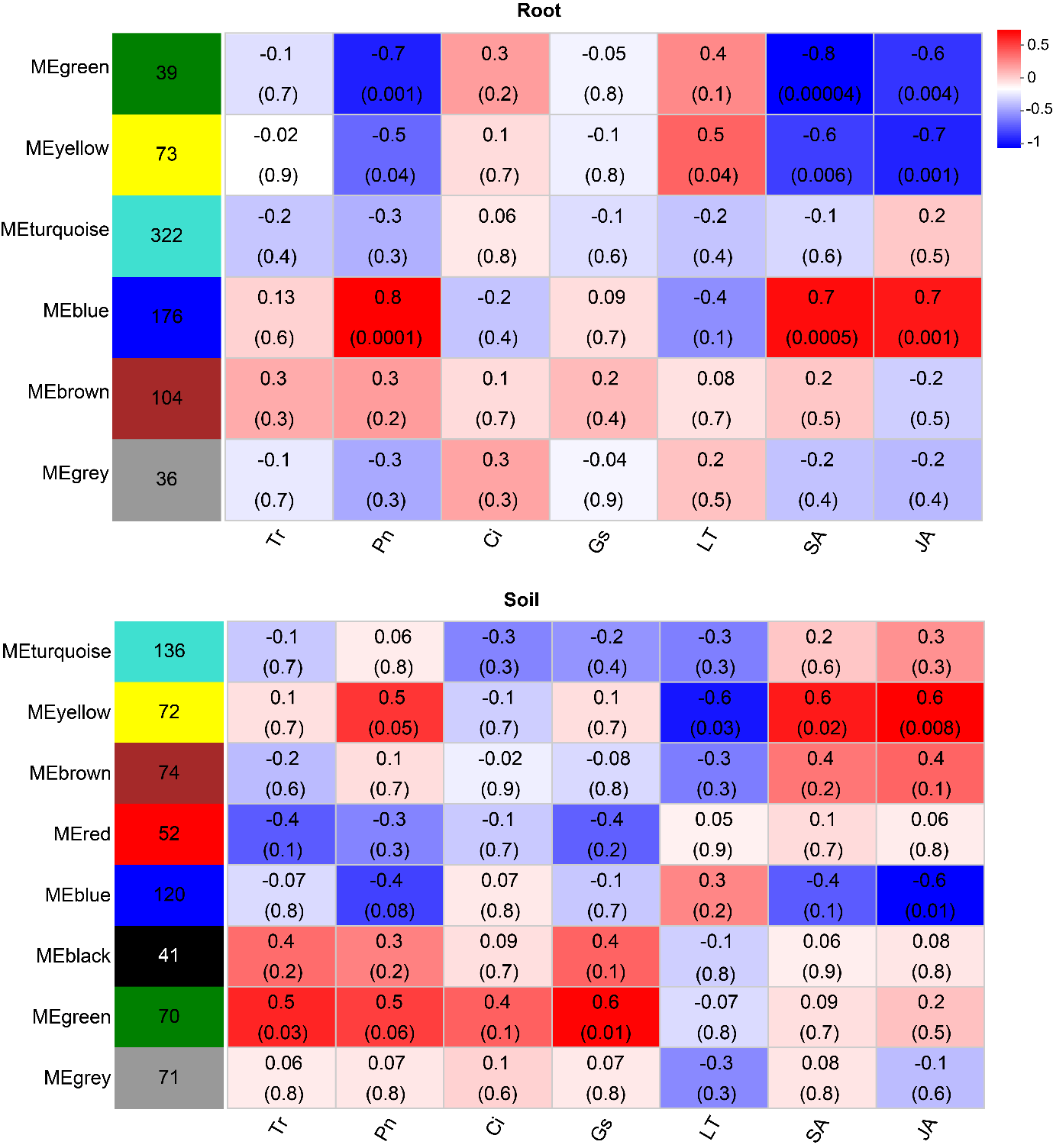


Figure S2. Weighted gene co-expression network analysis (WGCNA) of all DAMs (Differentially Expressed Metabolites) with FPKM (Fragments Per Kilobase of transcript per Million mapped reads) >1. The correlation among co-expression modules and physiological indicators. *Pn*, *Tr*, *Ci*, *Gs*, LT, SA, and JA represent the net photosynthetic rate, transpiration rate, and stomatal conductance, leaf thickness, salicylic acid, and jasmonic acid, respectively.


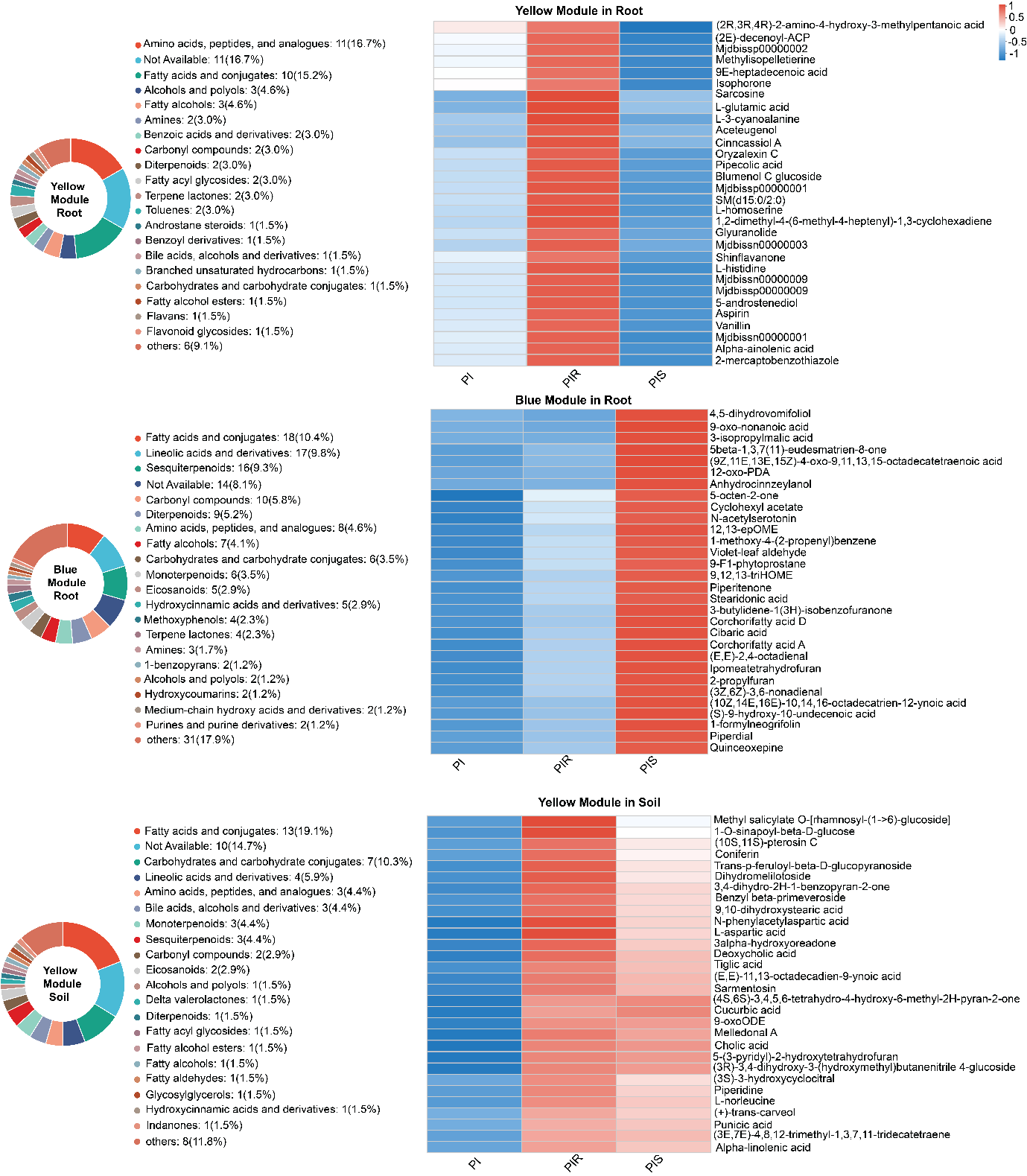


Figure S3. Heatmap of gene expression of the yellow module in roots, and the blue module in the rhizosheath soil. Pie chart displays counts of HMDB (Human Metabolome Database) taxonomy (subclass) for metabolites enriched in these modules. PI, PIR and PIS represent monocultured allelopathic rice with no barnyardgrass, allelopathic rice co-cultured with quinclorac-resistant and -susceptible barnyardgrass, respectively.


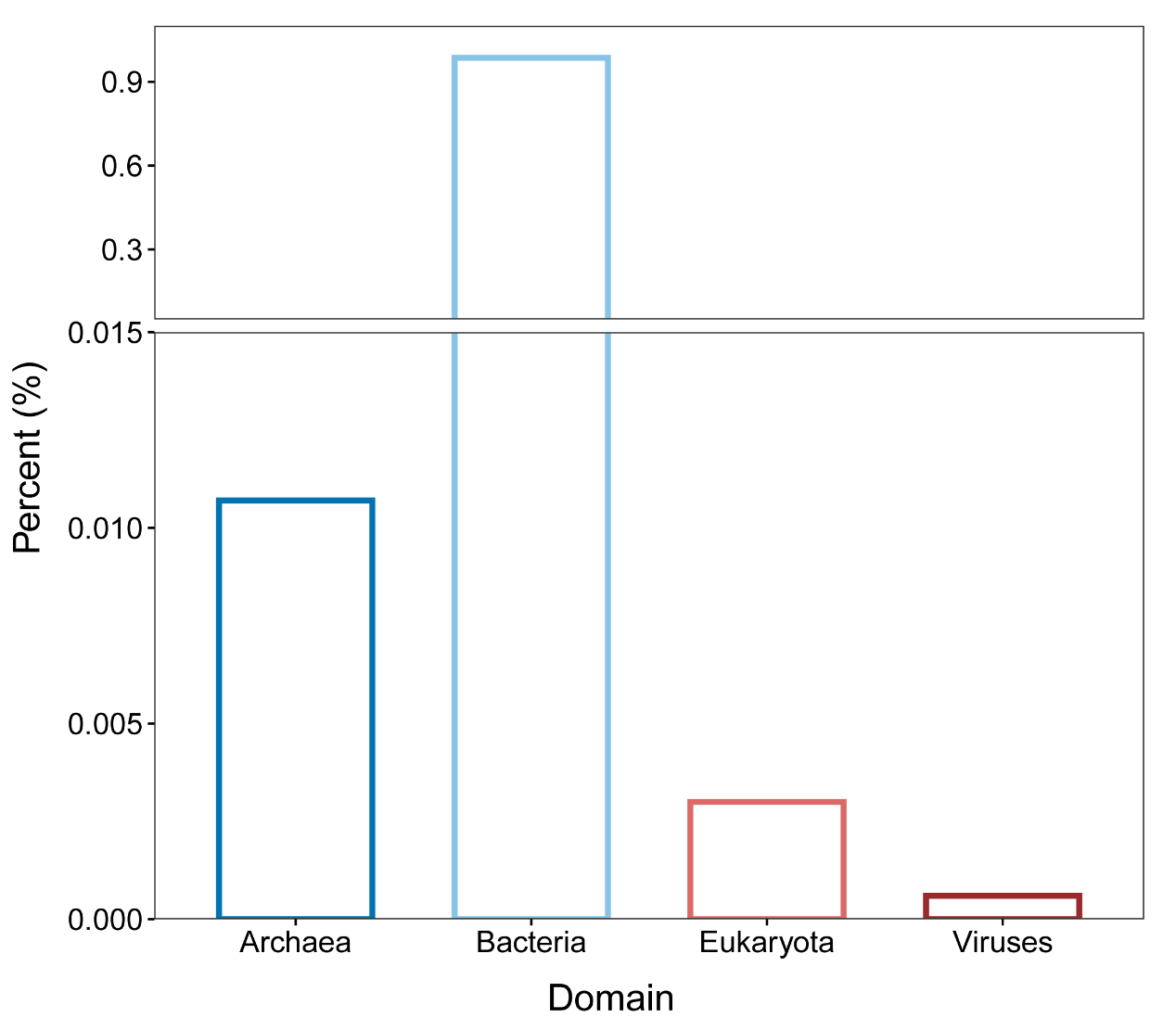


Figure S4. Taxonomic classification of allelopathic rice rhizosheath soil microorganisms across different domains and their proportional representation.


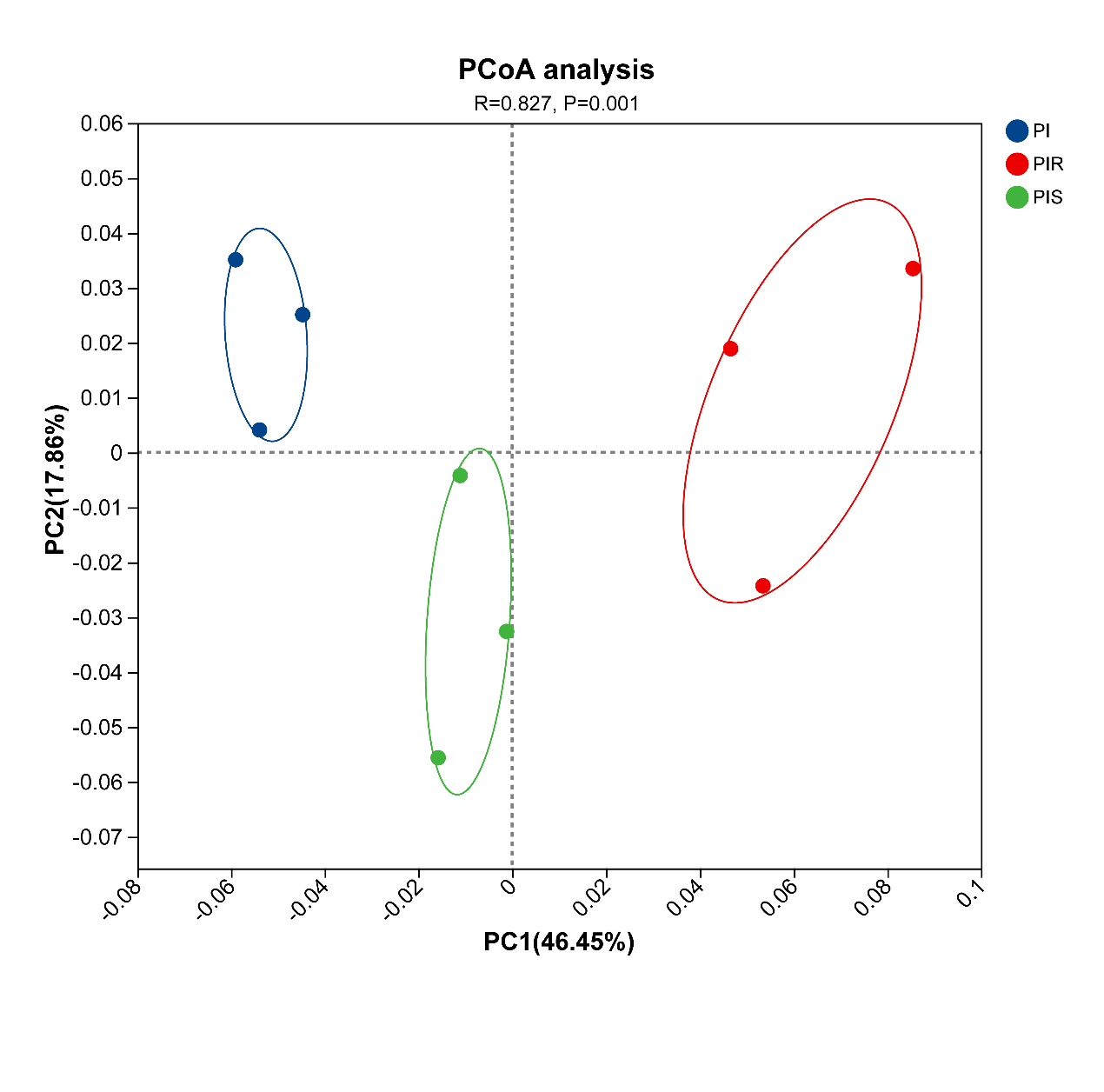


Figure S5. PCoA (Principal Coordinates Analysis) analysis based on Bray–Curtis distances at the genus level, demonstrating differentiation of allelopathic rice rhizosheath microorganisms in response to barnyardgrass stress. PI, PIR and PIS represent monocultured allelopathic rice with no barnyardgrass, allelopathic rice co-cultured with quinclorac-resistant and -susceptible barnyardgrass, respectively. Statistical differences among groups were assessed using ANOSIM with 999 permutations.


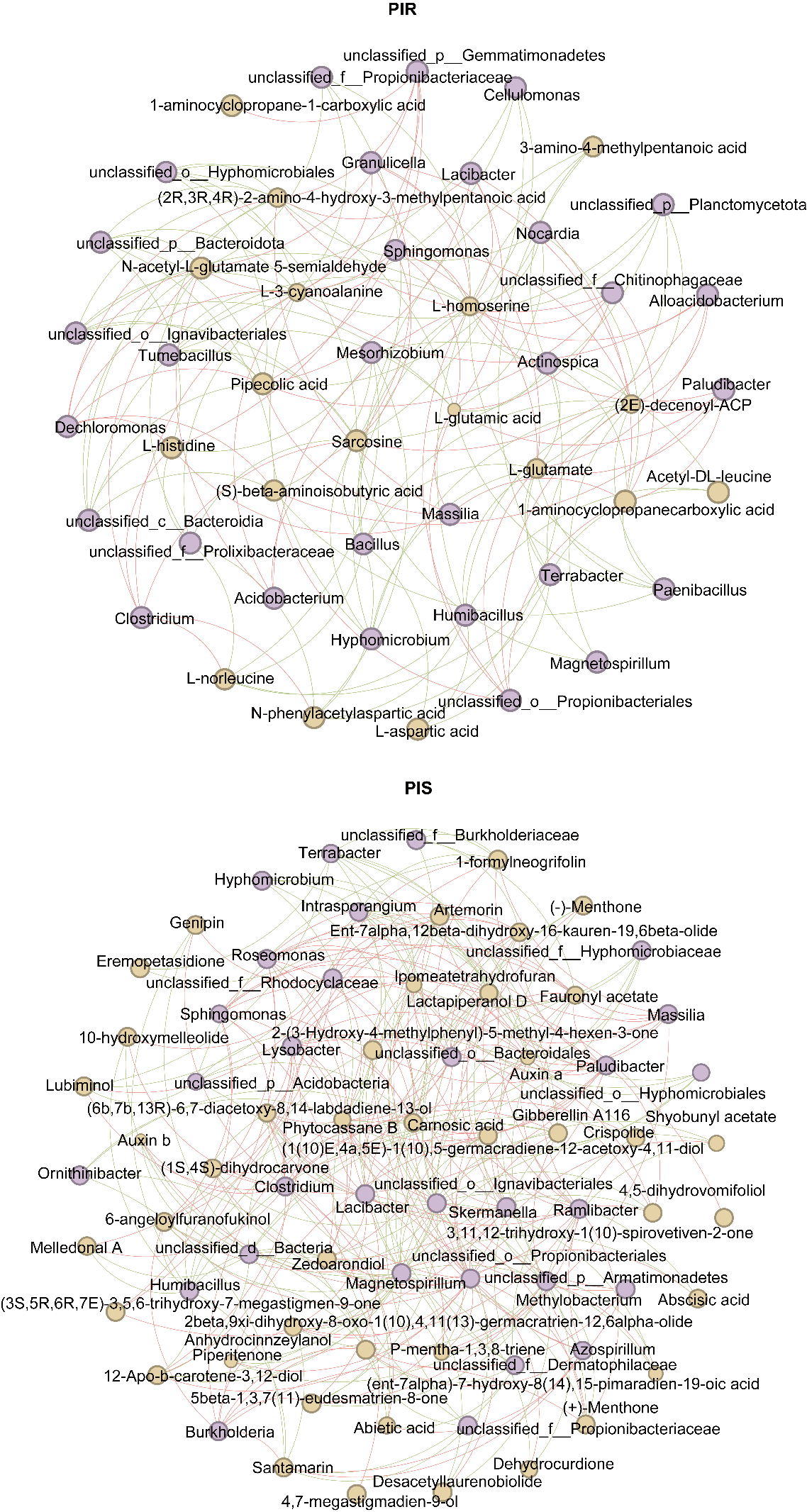


Figure S6. Correlation network diagram of bacteria and metabolites in barnyardgrass-stress rice at the genus level. Node size represents species abundance, with yellow and purple nodes representing metabolites and bacteria, respectively. The green and red lines indicate positive and negative correlations respectively, with line thickness reflecting the strength of correlation. A greater number of connecting lines indicates stronger connections between nodes.


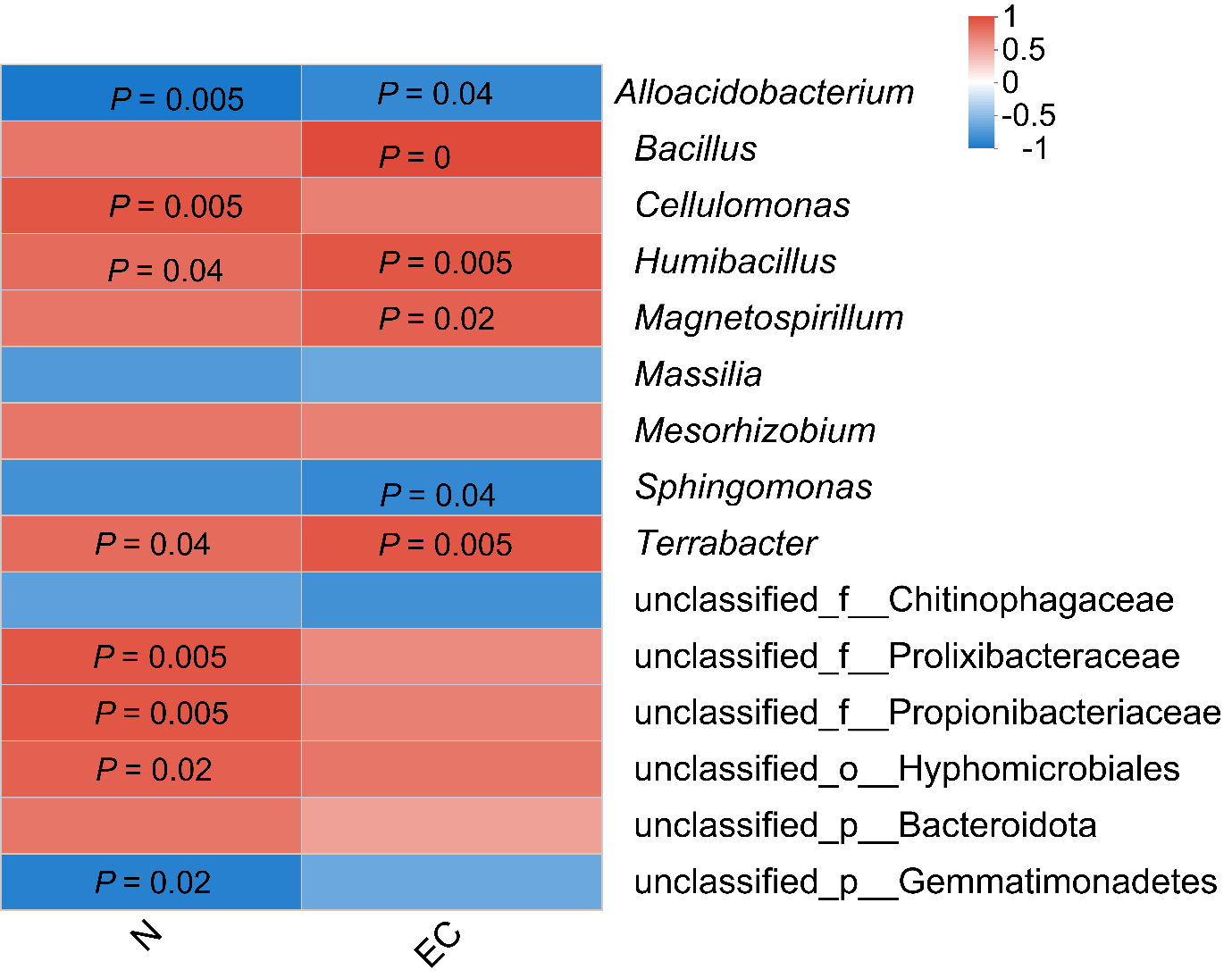


Figure S7. Spearman rank correlations between bacterial abundance and plant-available soil nitrogen, electrical conductivity (red and blue blocks indicate positive and negative correlation respectively).


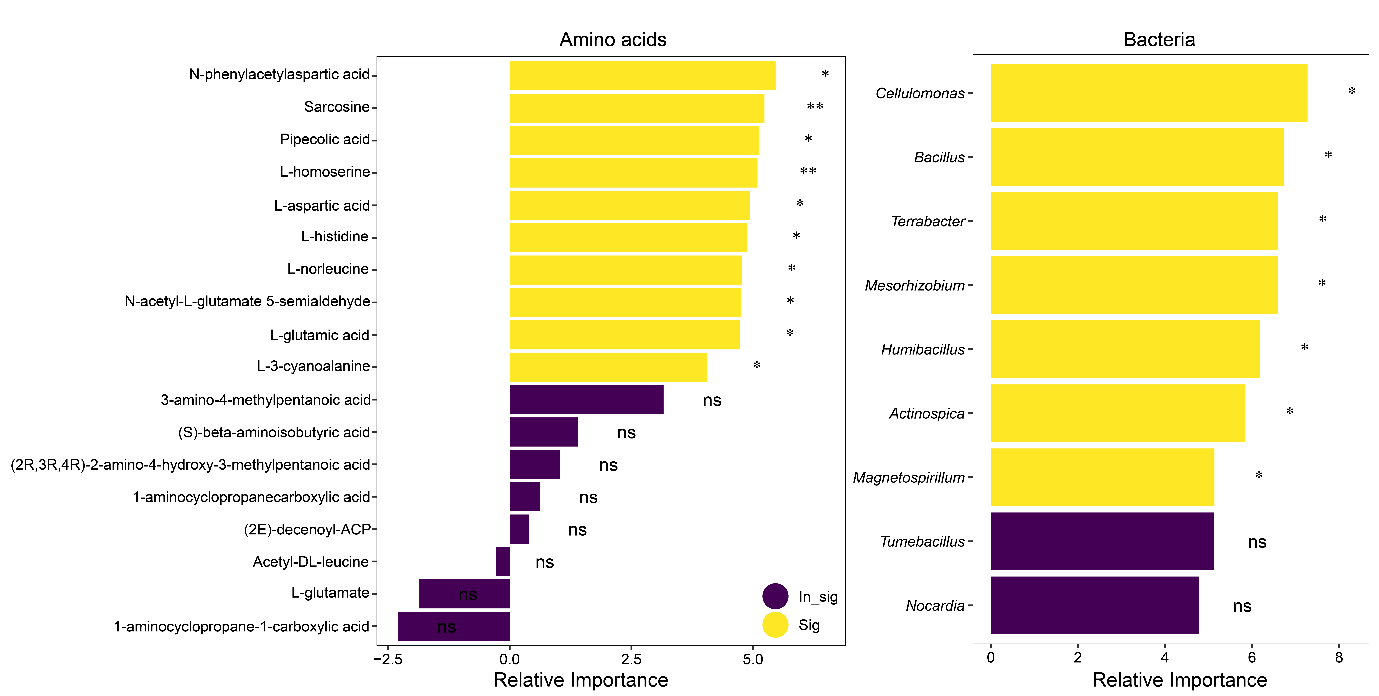


Figure S8. Random forest analysis demonstrating the significance of amino acids and bacteria in regulating plant-available soil nitrogen. The yellow bars represent amino acids (left panel) or bacteria (right panel) with statistically significant effects, while the purple bars indicate non-significant (ns) effects. * and ** denote *P*<0.05 and *P*<0.01, respectively. ‘ns’ stands for ‘not significant,’ meaning there was no statistically significant effect observed for those amino acids or bacteria.


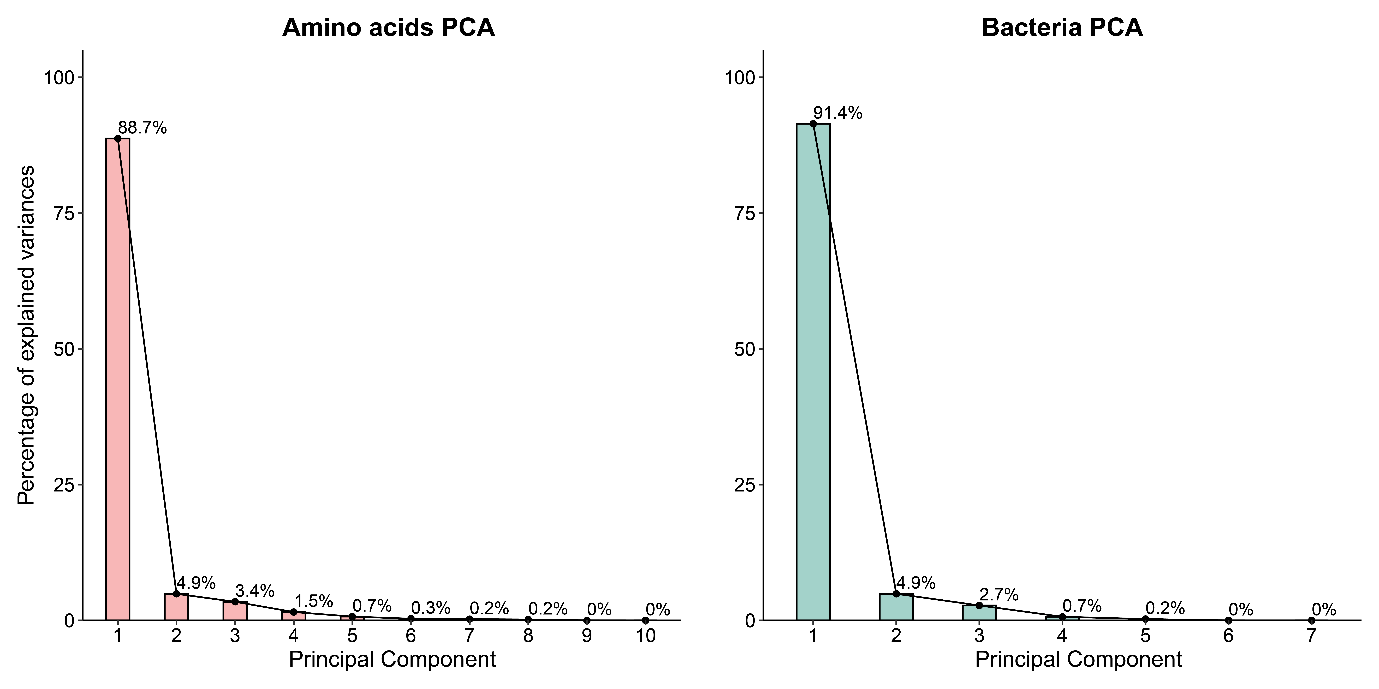


Figure S9. Principal component analysis (PCA) scree plots for amino acids and bacterial genera, showing the variance explained by each component.


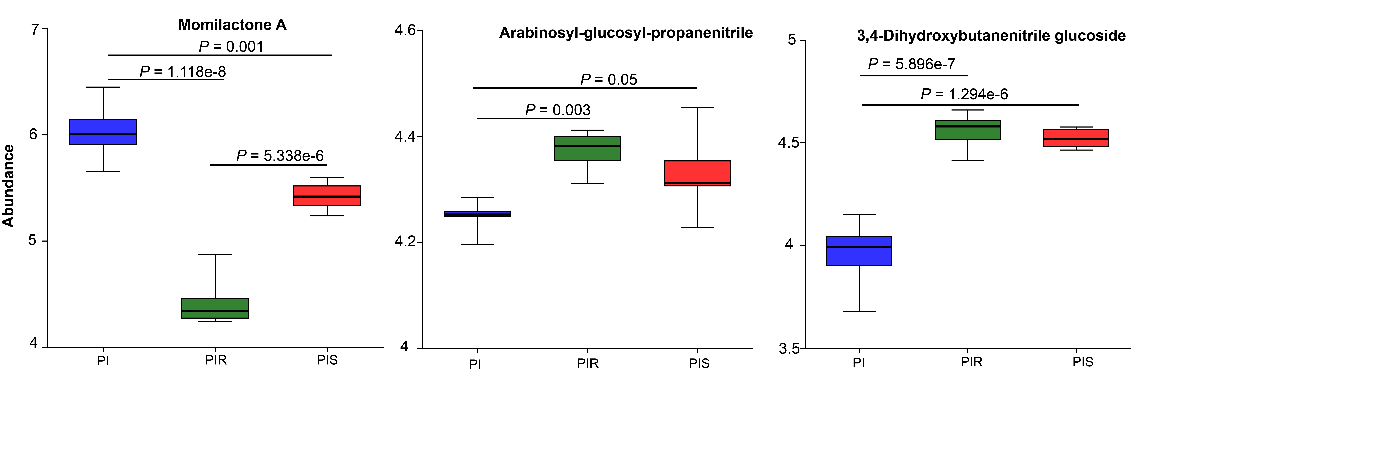


Figure S10. Abundance of momilactone A, and arabinosyl-glucosyl propanenitrile in the roots, 3,4-dihydroxybutanenitrile glucoside in the rhizosheath soil of the allelopathic rice co-cultured with barnyardgrass. PI, PIR and PIS represent monocultured allelopathic rice with no barnyardgrass, allelopathic rice co-cultured with quinclorac-resistant and -susceptible barnyardgrass, respectively.


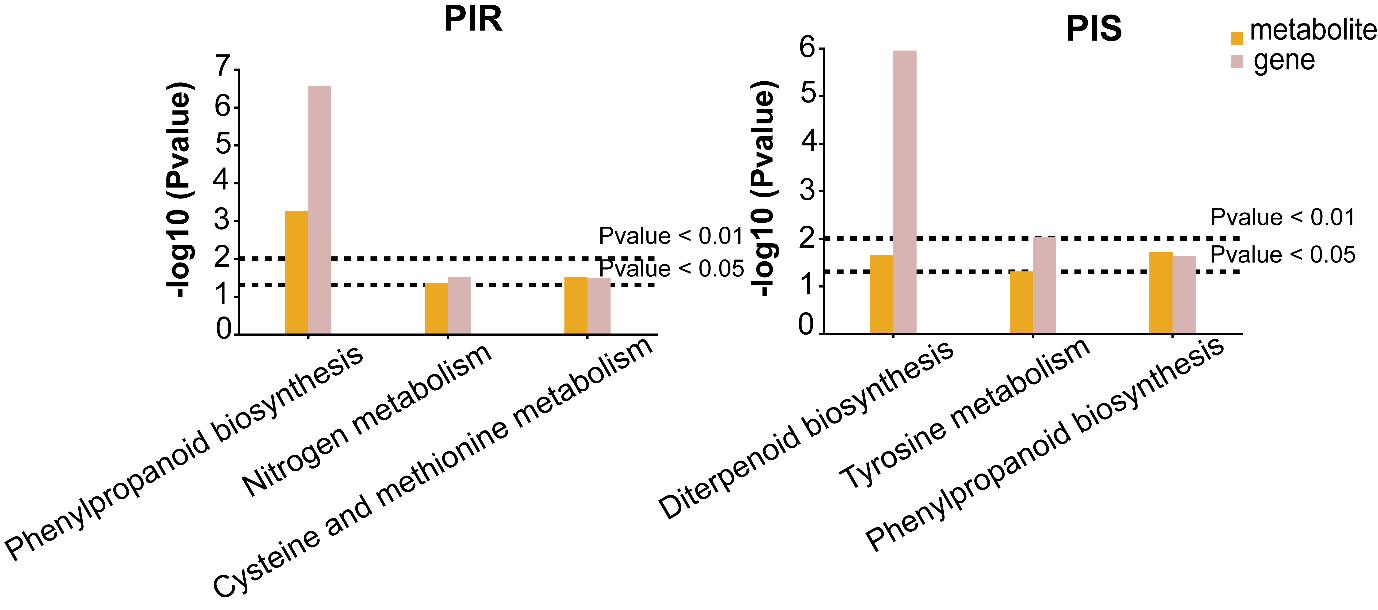


Figure S11. The *P* value for histogram of KEGG (Kyoto Encyclopedia of Genes and Genomes) enrichment analysis for integrated metabolomics and transcriptomics. PIR and PIS represent allelopathic rice co-cultured with quinclorac-resistant and -susceptible barnyardgrass, respectively.

The x-axis indicates the enriched metabolic pathways, and the y-axis represents -log (*P* value). Orange columns represent the enrichment *P* values of the differential metabolites, and pink columns, the enrichment P values of differentially expressed genes.


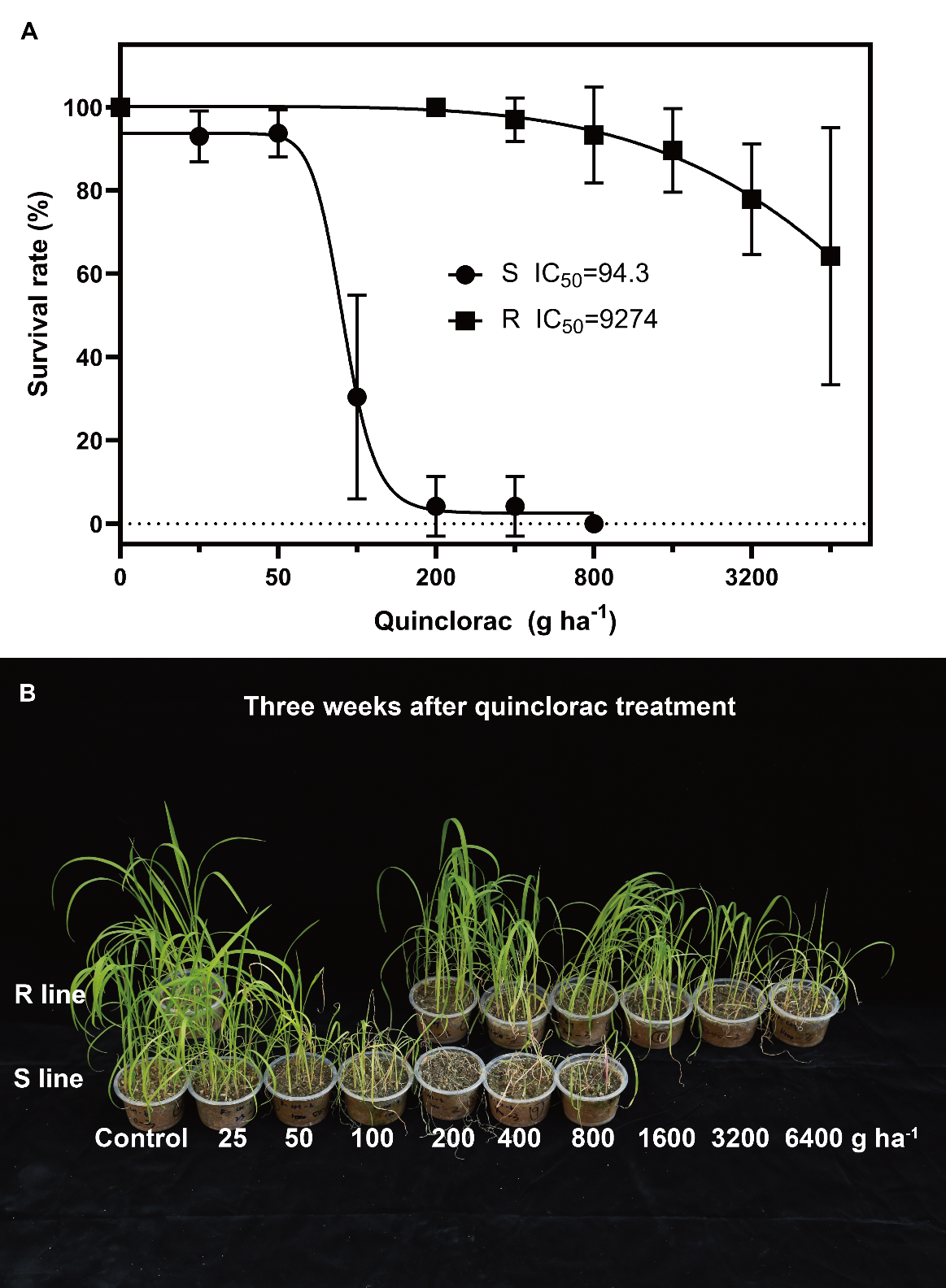


Figure S12. Dose response to quinclorac of barnyardgrass lines at three weeks after treatment.

(A) Dose-response curves showing survival rates of S and R lines following treatment with increasing rates of quinclorac. IC_50_ values were estimated based on survival three weeks after treatment.

(B) Representative phenotypes of S and R barnyardgrass lines three weeks after quinclorac application at different doses.
