# Supplementary material for: Distinct Defence Mechanisms of Allelopathic Rice Against Quinclorac‐Susceptible and ‐Resistant Barnyardgrass: Involvement of Specific Metabolites and Rhizosheath Microbiota
Source: Plant Biotechnol J. 2026 Feb 25;24(6):3876–96. doi: 10.1111/pbi.70611 (PMC13205853; doi:10.1111/pbi.70611)
Supplement: Supplementary file 1 — Table S1: Summary of trimming and read mapping results of the sequences generated from allelopathic rice root under PI, PIR and PIS treatments. Table S2: RT‐qPCR validation of the selected differentially expressed candidate contigs from the transcriptome dataset. Table S3: Rhizosheath microorganisms significantly increased in rice under resistant and susceptible barnyardgrass stress. Table S4: Representative KEGG orthologs (KOs) for quorum sensing and ABC transporter pathways and their leading bacterial taxa. Table S5: Bootstrap‐based (n = 10 000) variation partitioning analysis (VPA) results assessing the robustness of bacterial and amino acid contributions. Table S6: Amino acids used in the bioassays and their chemical information. Table S7: Differentially expressed auxin‐ and ethylene‐related genes in allelopathic rice under resistant (R) versus susceptible (S) barnyardgrass stress. Figure S1: Phenotypic comparison of flowering time between susceptible and resistant barnyardgrass grown under identical conditions. The resistant biotype shows earlier heading and flowering. Figure S2: Weighted gene co‐expression network analysis (WGCNA) of all DAMs (Differentially Expressed Metabolites) with FPKM (Fragments Per Kilobase of transcript per Million mapped reads) > 1. Figure S3: Heatmap of gene expression of the yellow module in roots, and the blue module in the rhizosheath soil. Pie chart displays counts of HMDB (Human Metabolome Database) taxonomy (subclass) for metabolites enriched in these modules. Figure S4: Taxonomic classification of allelopathic rice rhizosheath soil microorganisms across different domains and their proportional representation. Figure S5: PCoA (Principal Coordinates Analysis) analysis based on Bray–Curtis distances at the genus level, demonstrating differentiation of allelopathic rice rhizosheath microorganisms in response to barnyardgrass stress. Figure S6: Correlation network diagram of bacteria and metabolites in barnyardgrass‐stress rice at [file PBI-24-3876-s001.zip › Table S1.docx]

| Sample | Raw reads | Raw bases | Clean reads | Clean bases | Error rate (%) | Q20(%) | Q30(%) | GC content (%) |
| --- | --- | --- | --- | --- | --- | --- | --- | --- |
| PI | 42332206 | 6392163106 | 41780636 | 6282161154 | 0.0254 | 97.77 | 93.82 | 50.36 |
| PI | 52093836 | 7866169236 | 51426800 | 7741926972 | 0.0255 | 97.77 | 93.74 | 50.8 |
| PI | 41424834 | 6255149934 | 40889176 | 6150787049 | 0.0251 | 97.9 | 94.17 | 51.31 |
| PI | 43566152 | 6578488952 | 42872018 | 6444389974 | 0.0253 | 97.81 | 93.99 | 51.81 |
| PI | 52240192 | 7888268992 | 51555260 | 7752626956 | 0.0252 | 97.86 | 94.06 | 51.06 |
| PI | 41849880 | 6319331880 | 41335094 | 6220651348 | 0.0251 | 97.89 | 94.12 | 51.04 |
| PIR | 48261390 | 7287469890 | 47595034 | 7153134483 | 0.0251 | 97.91 | 94.2 | 50.84 |
| PIR | 53381244 | 8060567844 | 52669014 | 7922083023 | 0.0252 | 97.86 | 94.01 | 50.67 |
| PIR | 44844550 | 6771527050 | 44283028 | 6658606776 | 0.0253 | 97.83 | 93.91 | 50.47 |
| PIR | 51995570 | 7851331070 | 51180730 | 7689937919 | 0.0252 | 97.88 | 94.1 | 50.29 |
| PIR | 44133338 | 6664134038 | 43398808 | 6518145860 | 0.025 | 97.94 | 94.3 | 50.67 |
| PIR | 42381194 | 6399560294 | 41715244 | 6273590153 | 0.025 | 97.96 | 94.29 | 50.38 |
| PIS | 41718146 | 6299440046 | 41222282 | 6197032197 | 0.0254 | 97.82 | 93.88 | 50.5 |
| PIS | 55777994 | 8422477094 | 54949298 | 8272759256 | 0.0257 | 97.67 | 93.52 | 50.81 |
| PIS | 44833800 | 6769903800 | 44247600 | 6651115874 | 0.025 | 97.94 | 94.26 | 51.1 |
| PIS | 45483886 | 6868066786 | 44817554 | 6733350306 | 0.0251 | 97.88 | 94.18 | 51.44 |
| PIS | 50136500 | 7570611500 | 49417602 | 7433459457 | 0.0253 | 97.82 | 93.97 | 51.46 |
| PIS | 47873486 | 7228896386 | 47249570 | 7107231177 | 0.0252 | 97.85 | 94 | 51.03 |

Table S1. Summary of trimming and read mapping results of the sequences generated from allelopathic rice root under PI, PIR and PIS treatments. PI, PIR and PIS represent monocultured allelopathic rice with no barnyardgrass, allelopathic rice co-cultured with quinclorac-resistant and -susceptible barnyardgrass, respectively.
